# Supplementary material for: aurora: a machine learning gwas tool for analyzing microbial habitat adaptation
Source: Genome Biol. 2025 Mar 23;26:66. doi: 10.1186/s13059-025-03524-7 (PMC11930000; doi:10.1186/s13059-025-03524-7)
Supplement: Supplementary file 1 — Additional file 1: Supplementary text and figures – Detailed explanation of aurora algorithm. Additional results and discussion [127–156]. [file 13059_2025_3524_MOESM1_ESM.docx]

# Supplementary Materials: *aurora*: A Machine Learning GWAS Tool For Analyzing Microbial Habitat Adaptation

# SUPPLEMENTARY FIGURES


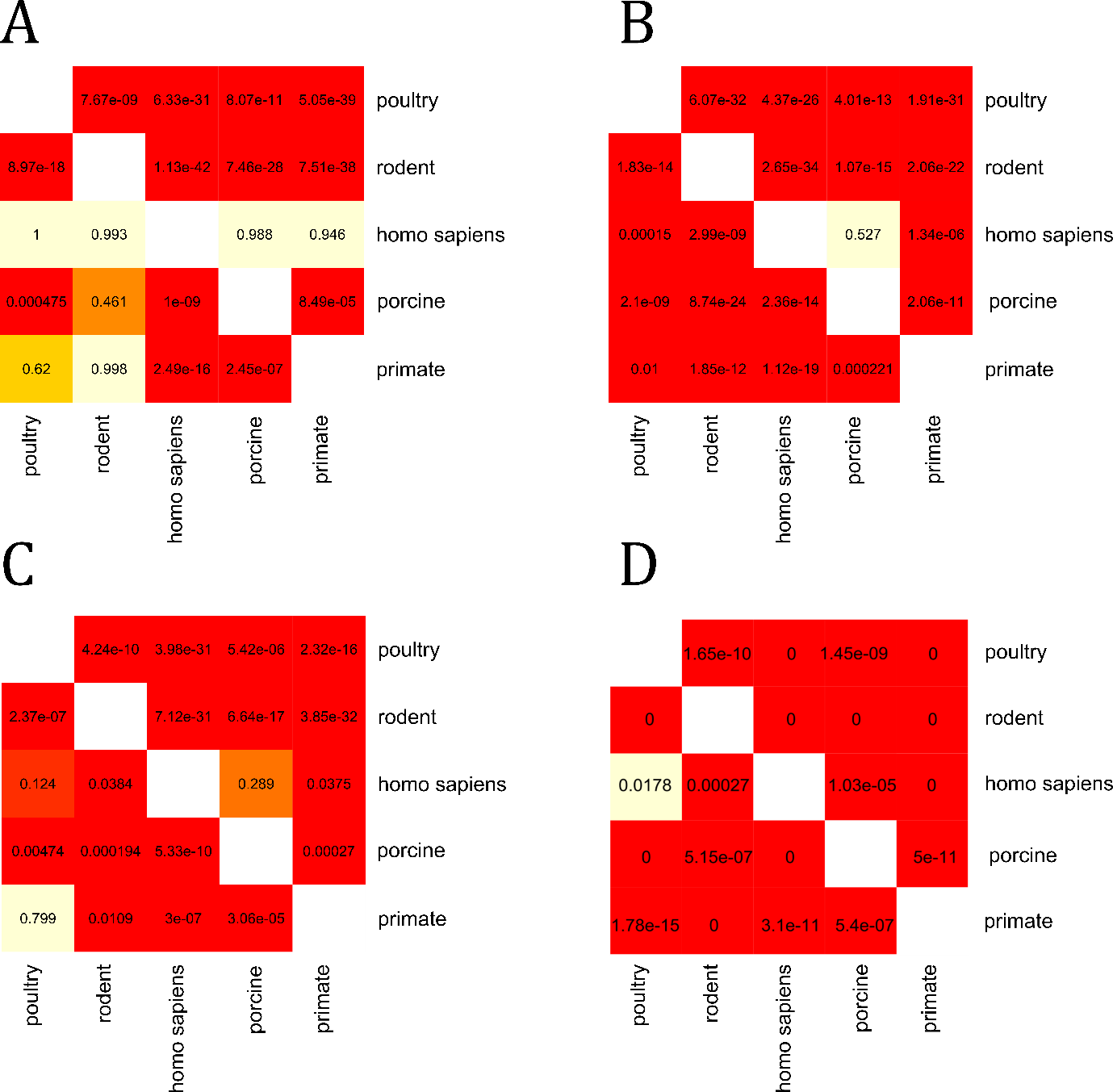


**Figure S1.** Results of Threshold Calculation Phase. Each matrix shows the result of multiple pairwise Kolmogorov-Smirnov tests for one ML algorithm. The test examines if the classification probabilities of strains that were mislabeled are lower than strains that were not mislabeled. For example, a *p*-value in 1^st^ row and 2^nd^ column indicates if strains mislabeled from rodent class to poultry class have lower poultry classification probability than non-mislabeled poultry strains. If the species is uniquely adapted to rodents and poultry then *p*-values in at least one cell ([1,2] or [2,1]) should be below 0.05. A) results from Random Forest, B) results from AdaBoost, C) results from log regression, and D) results from CART.


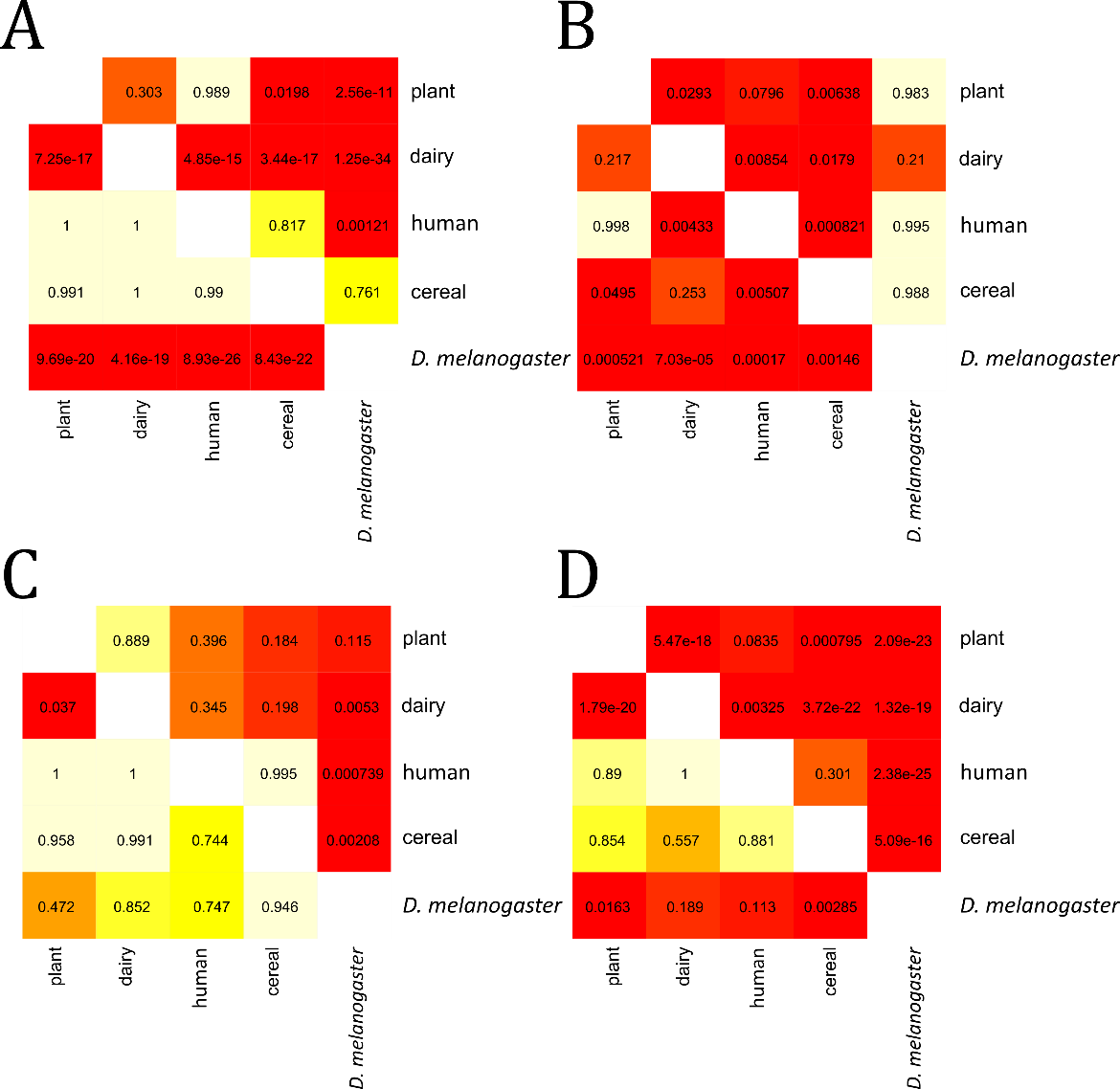


**Figure S2.** *p*-value matrix of *Lactiplantibacillus plantarum* dataset. Each matrix shows the result of multiple pairwise Kolmogorov-Smirnov tests for one ML algorithm. The test examines if the classification probabilities of strains that were mislabeled are lower than strains that were not mislabeled. For example, a *p*-value in 1^st^ row and 2^nd^ column indicates if strains mislabeled from dairy class to plant class have lower plant classification probability than non-mislabeled plant strains. If the species is uniquely adapted to dairy and plants then *p*-values in at least one cell ([1,2] or [2,1]) should be below 0.05. A) results from Random Forest, (B) results from AdaBoost, C) results from log regression, and D) results from CART.


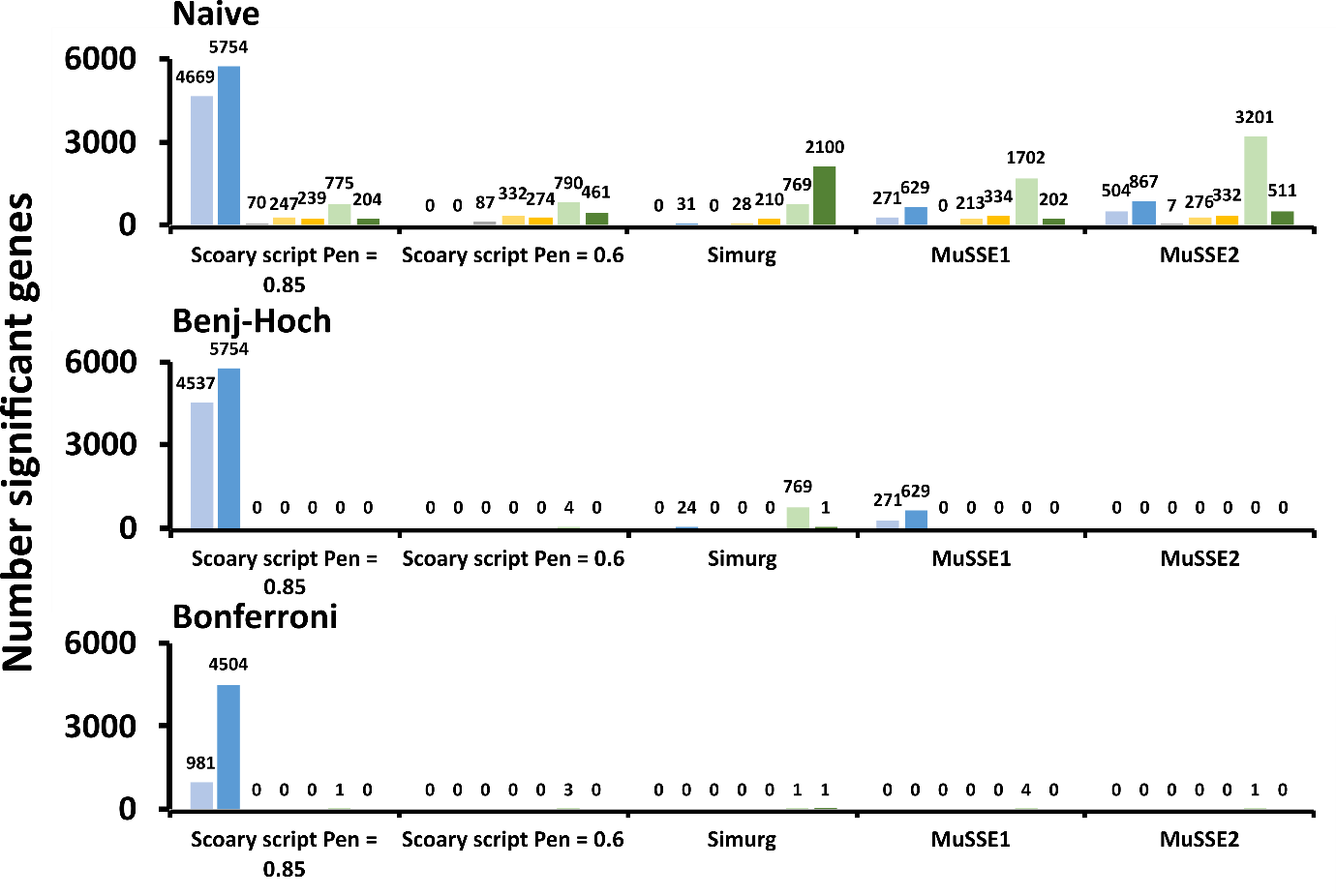


**Figure S3.** In this analysis, the phenotype labels of the five simulated datasets were randomized and the graphs show the number of significant (false positives) GFs that each test produced. The first graph shows naïve results – no multiple tests adjustment (same graph as Fig 3C in the main text) and the two remaining graphs show the same results but adjusted with Bonferroni adjustment or Benjamini-Hochberg (FDR) method.


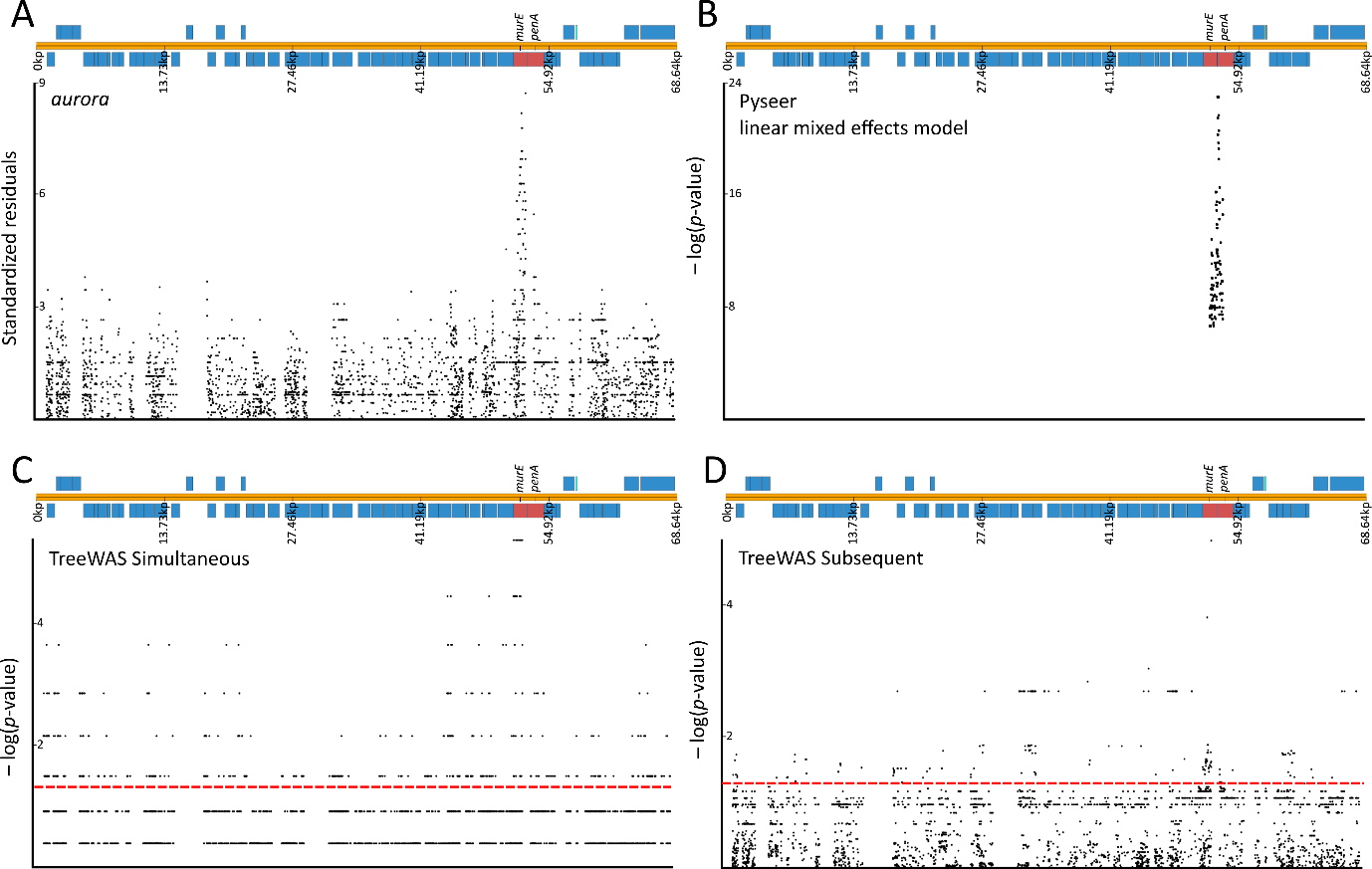


**Figure S4.** Manhattan plots showing associations of variants conferring penicillin resistance in *Neisseria meningitidis*. A) Results of *aurora* analysis with core genome SNPs, B) results of the analysis with unitigs using linear mixed effects model implemented in Pyseer (only significantly associated unitigs are shown), C) results of SNPs analysis with Simultaneous test implemented in TreeWAS, and D) results of SNPs analysis with Subsequent test implemented in TreeWAS. The red lines indicate naïve significance threshold (*p*-value = 0.05). This figure shows that just as other tools *aurora* can clearly identify loci associated with antibiotic resistance (*murE* and *penA*) and its functionality is thus not limited only to microbe-habitat associations.


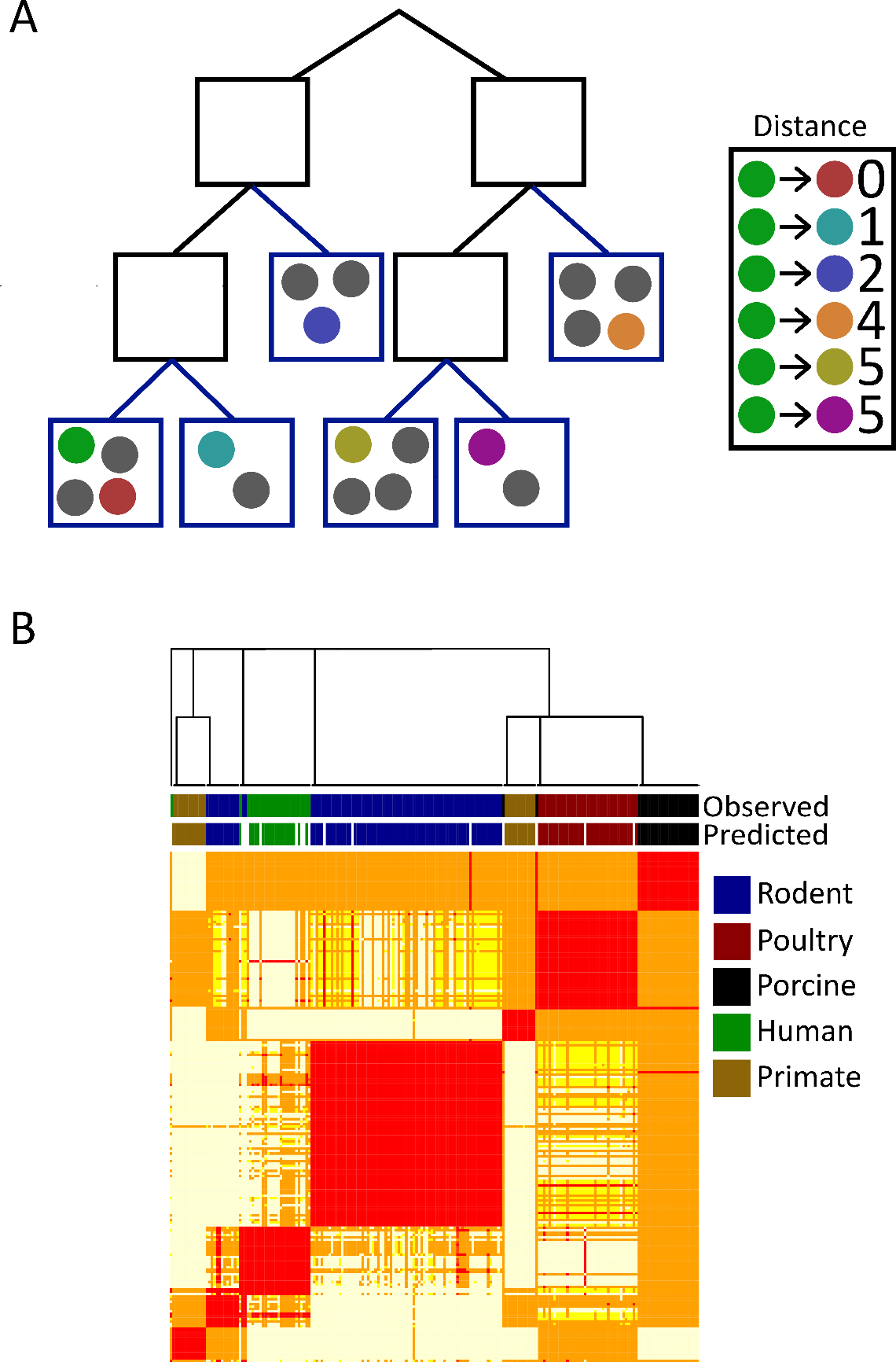


**Figure S5.** Distance measure based on CART models. A) Description of the distance measure calculated from CART model. The hypothetical tree shows a CART model with six leaf nodes. The colored circles represent strains. On the right, a distance measure of the green point to six other points is depicted. B) Distance matrix of *L. reuteri* strains based on the CART proximities.


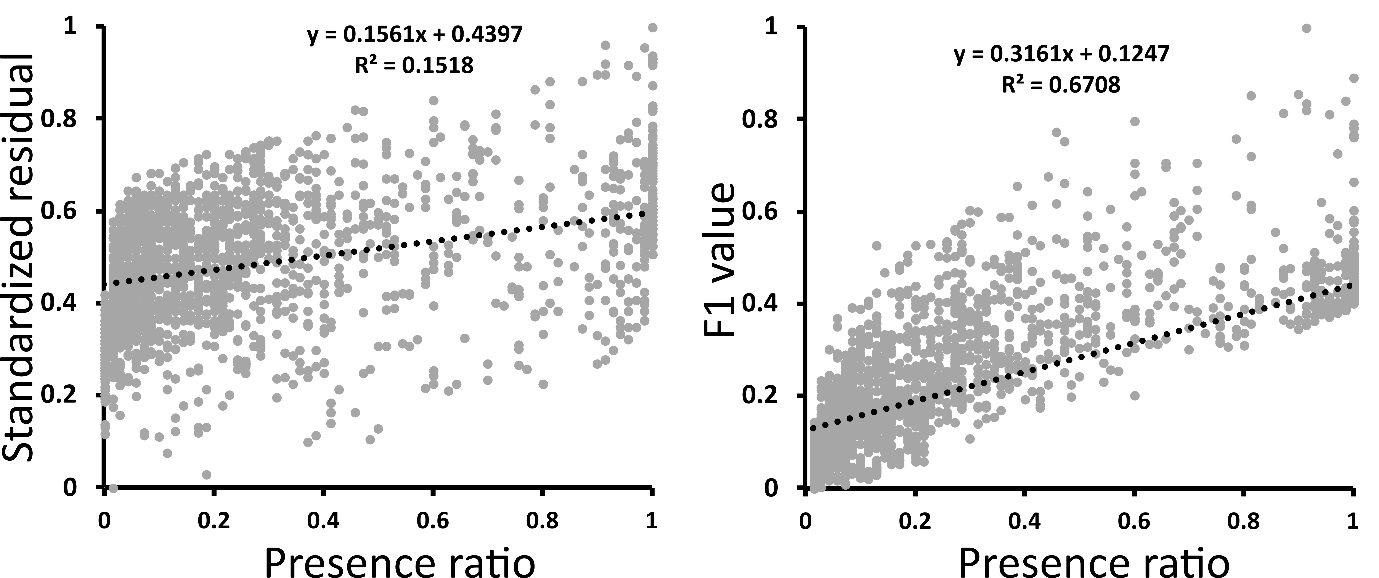


**Figure S6.** The relationship between standardized residual or F1 value and presence ratio $\frac{a}{N}$ for each GF in *L. reuteri* pangenome. *a* is the number of rodent isolates in *L. reuteri* dataset where the GF is present, and *N* is the total number of rodent isolates. A linear regression model was fitted to both datasets and as evident from the slopes, F1 values tend to increase more with increasing presence ratio.


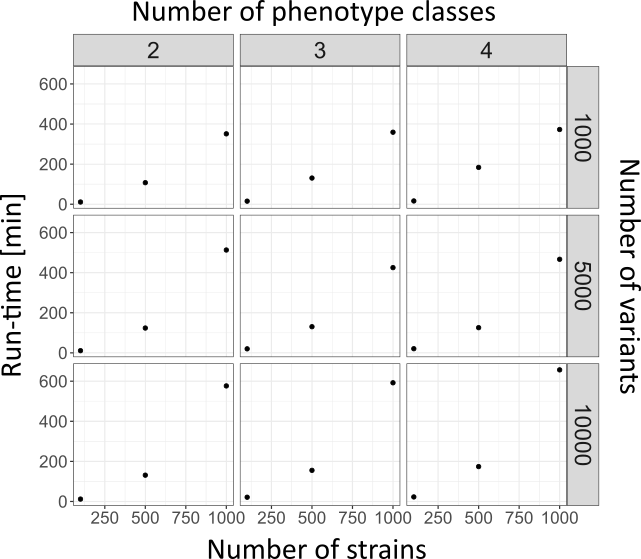


**Figure S7.** The run-time depends on the number of variants (before default filtering), the number of phenotype classes, and the number of strains. Among these, the number of strains has the greatest impact on run-time, with approximately exponential scaling. The datasets used for this simulation were generated in the same way the MuSSE1 dataset.

# DESCRIPTION OF THE TOOL

Microbial Genome-Wide Association Studies (mGWAS) have been thoroughly reviewed elsewhere [1,2]. In brief, mGWAS is a set of methods used to identify genetic variations associated with phenotypic traits in microbial populations. It involves analyzing the genomes of a large number of microbial organisms. *aurora* was specifically developed to analyze habitat adaptations, but it can be used for the analysis of any categorial phenotype. Our validation described in the main text of this study indicates that *aurora* should outperform other tools especially where there is a possibility that class labels were assigned incorrectly, in cases where the phenotype can be explained by more than one genetic mechanism, or when the analyzed phenotype is not transient.

Input objects

Three input objects are required by *aurora*. The first is a binary matrix (R data frame or matrix) that shows the presence/absence of features in analyzed strains. *aurora* can use the direct output from pangenome tools Roary [106] and Panaroo [107]. Alternatively, the user can provide a system path to the data generated by DRAM [110], fsm-lite (<https://github.com/nvalimak/fsm-lite>), unitig-counter (<https://github.com/bacpop/unitig-counter>), or a vcf file that contains core-genome SNPs (either bi-allelic or multi-allelic). Additionally, any binary matrix can also be used provided that it is formatted correctly. SNPs, unitigs, and k-mers are more numerous features than genes in a pangenome matrix (the number of genes in the pangenome is usually between 5,000 and 30,000 while there can be millions of unique SNPs/k-mers in the analyzed population) thus more stringent pre-filtering should be applied. *aurora* provides parameters to reduce the number of features and preserve only those that are informative.

The second object to be supplied is a matrix or data frame where the first column contains unique strain indices (these should start with a letter and contain only letters, numbers, and underscores “_”), and the second column contains the phenotype of each strain (argument pheno_mat). Multiple categories (*syn*. classes) per phenotype are supported. It is strongly recommended that the phenotype has at least 20 strains per class assuming that the strains are not clonal. The last required input is either a phylogenetic tree that contains all the analyzed strains or a distance matrix (i.e., Mash distance [103], Hamming distance, Jaccard distance, ANI, or cAAI matrix) that represents a phylogenetic distance between the strains. If cAAI or ANI matrices are used, the values must be converted into distances. By default, *aurora* runs with all four machine learning (ML) algorithms (see below). Thus, for medium-size datasets (100-500 strains) and 3-5 phenotypes, the analysis takes several hours on a standard desktop machine. However, the time can be significantly reduced by choosing only some ML algorithms, selecting random_walk over phylogenetic_walk, or by reducing the values of bag_size and no_rounds arguments.

Output objects

The *aurora* algorithm is a two-step process. First, *aurora* determines if any adaptation towards the phenotype exists, it identifies strains that belong to a different class from their recorded phenotype (“mislabelled” strains) and strains without adaptation to any class of the phenotype. This is governed by the function aurora_pheno(). Secondly, *aurora* removes these strains and identifies genes (or any user-supplied features) that are responsible for the adaptation to the phenotype – a process performed by function aurora_GWAS().

The function aurora_pheno() produces a matrix for each machine learning algorithm that was used. The matrix values are pair-wise comparisons calculated by a two-sample Kolmogorov-Smirnov test. The test measures the likelihood that we would see these two sets of samples if they were drawn from the same population. If an adaptation towards class A and B exists, then the two phenotypes should have at least one *p-*value – P(A|B) and P(B|A) – below 0.05. If both *p-*values are above 0.05 then the two classes should be considered interchangeable and thus there is no genetic adaptation towards the two classes. These could be grouped in the next run of *aurora*. aurora_pheno() also produces a table for each machine learning method used. This table contains the predicted phenotype class for each strain. Additionally, the function outputs a table showing feature importance values and summary figures. Before fitting the ML models aurora_pheno() reduces or collapses features using numerous filters. The number of filtered features is returned. If argument fit_parameters is set to TRUE then aurora_pheno() fits best hyperparameters for AdaBoost and Random Forest. These hyperparameters are also part of the output list.

The next step is governed by the function aurora_GWAS(). The required input is the same as for aurora_pheno(). Function aurora_GWAS() can be run regardless of whether aurora_pheno() was run beforehand or not. If results from aurora_pheno() are available, then strains, where the predicted phenotype does not match the observed, are removed. Additionally, strains that have a low classification probability for their observed phenotype can also be removed. Subsequently, the remaining set of strains is adjusted for population structure, and standardized residuals (calculated from χ² test), precision, recall, and F1 statistics are calculated for each feature using the adjusted sample. The output is a feature table that contains the values for the four statistics and a presence/absence ratio in the adjusted sample. The features that are the most significant for each class should have the highest standardized residual and F1 statistic. In the following, we present how *aurora* works using a *Limosilactobacillus reuteri* dataset as an example. We also explain design and parameter choices and how *aurora* can be customized to fit the needs of any user.

## Example dataset

In this example, we use an *L. reuteri* dataset comprising 207 genomes downloaded from the NCBI assembly database in January 2023. *L. reuteri* is a gut commensal bacterium that has co-evolved with its mammalian hosts over millions of years [127]. The bacterium has developed various adaptations to survive in the host environment [66,128,129]. This species diversified into multiple host-associated lineages [16] and it is considered a model system for studying microbial adaptation to vertebrate gut. Adaptation to five hosts will be analyzed: poultry (39 strains), rodent (90 strains), *Homo sapiens* (27 strains), porcine (26 strains), and primate (25 strains). The genomes were first annotated by PROKKA [114] and then Panaroo [106] was used to construct the pangenome. The pangenome contained 8,534 gene families (GFs) in total with 1,200 core genome GFs and 6,186 GFs present in the cloud genome (present in less than 15% of all strains).

## Filtering the input features

First, *aurora* removes rare features (low_perc_cutoff = 3, features present in less than 3% of all analyzed strains) and abundant features (upp_perc_cutoff = 99, features present in more than 99% of all analyzed strains). If the number of features after this initial filtering is still large (> ~10,000) we recommend setting run_chisq = TRUE. This will run the χ² test with the presence/absence pattern of every feature. By default, if the *p*-value from the χ² test is > 0.1 (parameter cutoff_chisq = 0.1) the feature is removed. In the example here we left low_perc_cutoff and upp_perc_cutoff at their default values and set run_chisq to FALSE. The filter removed 5,308 GFs and left 3,226 GFs for further analysis.

Next, the user has a choice to apply the ancestral reconstruction filter (parameter: ancest_rec_filter). The purpose of this step is to remove GFs that were gained and lost multiple times during the evolution. These GFs are often associated with mobile genetic elements responsible for or associated with horizontal gene transfer *i.e.,* transposases, toxin-antitoxin systems, or mobility genes necessary for conjugation. These GFs are the most abundant genes in nature [130,131]. They alone however do not indicate adaptation to a habitat. A group of strains without host adaptation can possess the same transposases and *aurora* could falsely assign those as autochthonous strains. Because these elements have variable sequences and different functional annotations, *aurora* does not remove them explicitly. Instead, *aurora* first calculates ancestral state reconstruction using package ape [132] which implements the Wagner parsimony method [133] for ancestral state reconstruction. Subsequently, a value *y* is calculated for each GF as:

$$\gamma= \log\frac{o}{\sum_{i=1}^{o} \sum_{k=1}^{j} x_{i,k}}$$

Equation 1

Where *o* (origin) is the number of times a GF was acquired in the phylogeny, *j* is the number of edges *x* (edge length) that leads from origin *o^i^* to all tip nodes of the phylogenetic tree. Then a z-score is calculated for all *γ* values and GFs with a z-score higher than parameter cutoff_asr (default 2) are removed. The distribution of z-scores is shown in Fig S8 and it roughly follows normal distribution. We annotated all 119 *L. reuteri* GFs (Additional file S8) that were removed by ancestral reconstruction filter and the results confirmed that many of the removed GFs are related to horizontal gene transfer. However, the results also show that some of these GFs are present in all *L. reuteri* strains like DNA replication proteins, RNA polymerase subunits, and various metabolic enzymes – these should have been merged with GFs in the core genome. Many of these GFs also lacked annotation. We hypothesize that these GFs are gene fragments that were not detected by Panaroo, a result of imperfect genome assembly or incorrect gene calling. In either case, these GFs are not important for host adaptation and should be removed. The hyperparameter cutoff_asr should be carefully examined if the number of removed features is too high (~10% of all features). We do not advise setting the parameter below 2 as that can result in the removal of features associated with the analyzed phenotype. Since this step uses a phylogenetic tree, it cannot be run if a phylogenetic distance matrix is supplied instead.


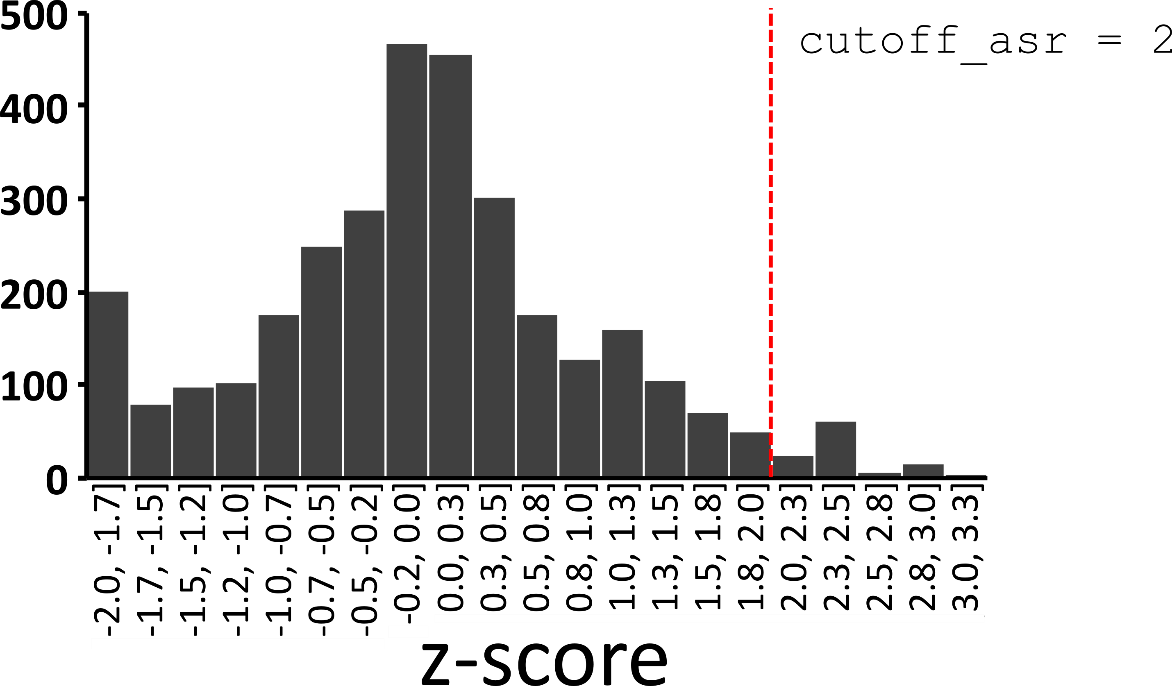


**Figure S8.** Histogram of distribution of z-scores calculated from *γ* (Equation 1) values used in ancestral reconstruction filter. GFs with a z-score higher than hyperparameter cutoff_asr (default: 2) are removed.

In the next step, highly correlated GFs are grouped. This step can be omitted but it is highly recommended to run it. In logistic regression, multicollinearity between the features can lead to unstable and unreliable estimates of the coefficients. In Random Forest, highly correlated features can lead to biased variable importance measures, where one of the correlated features may be deemed more important than the other due to chance. When AdaBoost or CART trees are used, highly correlated features can lead to overfitting. In practice, there are many GFs whose presence/absence pattern is correlated. A typical example are genes in operons or plasmids. There are two methods implemented in *aurora* that facilitate grouping of highly correlated features: jaccard_filter and hamming_filter. By default, hamming_filter is used. Both methods cannot be run together. In jaccard_filter, Jaccard distance is first calculated as

$$d\left( {GF}_{a},{GF}_{b} \right)=1-\frac{\left| {GF}_{a}\cap{GF}_{b} \right|}{\left| {GF}_{a}\cup{GF}_{b} \right|}$$

Equation 2

Where *d()* is a Jaccard distance, *GF_a_* and *GF_b_* are gene families *a* and *b*. The resulting Jaccard distance matrix then serves as an input into DBSCAN algorithm [134]. *aurora* uses the package dbscan for this task [111]. DBSCAN has two hyperparameters eps_val (radius of the epsilon neighborhood) and minPts_val (number of minimum points required in the eps neighborhood for core points). We do not advise modifying minPts_val (default 3) as this is the smallest cluster size. On the other hand, it might be necessary to modify eps_val (default 0.01) if the algorithm over/under clusters. This hyperparameter choice was suitable for most datasets analysed herein. Running this filter on the *L. reuteri* dataset resulted in grouping 797 GFs into 169 new GFs. Amongst those grouped GFs, there was also an *ure* operon known to be essential for adaptation to the rodent gut [87]. Users should check if the grouping was correct by looking at the number of grouped GFs and also by visually inspecting the MDS plot of the Jaccard distance matrix (Fig S9). The gray color represents GFs that were not grouped. Other colors in the plot show GFs that were collapsed into one GF. Multiple collapsed GFs have the same color since the number of colors is limited. The user should confirm that the newly grouped GFs are evenly distributed compared to the overall distribution (*e.g.,* more dense regions have more grouped GFs). The plot should also show that grouped GFs are mostly small ellipsoid clusters or appear as single dots (due to the number of GFs visualized).


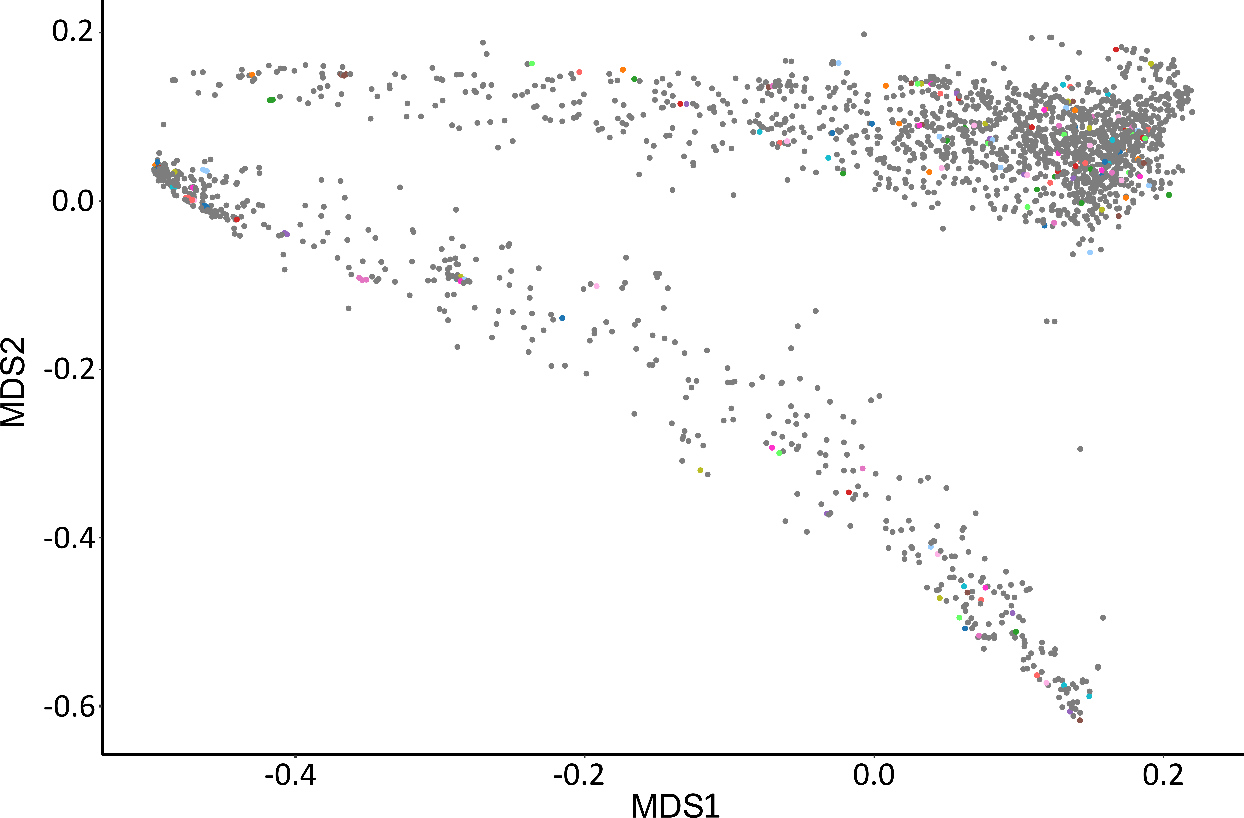


**Figure S9.** Visualization of pairwise Jaccard distance matrix of GFs in the *L. reuteri* dataset. Multidimensional scaling (MDS) was used to reduce the number of dimensions to two. The gray color shows GFs that were not grouped by jaccard_filter. Other colors show GFs that were collapsed. The colored dots represent at least 3 individual GFs that were grouped. The colors are not unique for each GF. Thus, multiple collapsed GFs can have the same color.

Since jaccard_filter might require fine-tuning of the eps_val hyperparameter an alternative and also default grouping method is hamming_filter. This grouping algorithm clusters only features that have less or equal number of mismatching positions than hamming_cutoff (default 3). The mismatching position is a case when *GF_a_* is present in *strain_x_* and *GF_b_* is absent in *strain_x_* or vice versa. The first step of hamming_filter is calculating Hamming distance matrix between all features. Then *aurora* loops through all GFs and if a *GF_a_* has Hamming distance <= hamming_cutoff to *GF_b_*, *GF_c_* … *GF_n_* then a submatrix *A* of all these GFs is extracted. In *A,* any *GF_x_* has Hamming distance <= hamming_cutoff to *GF_a_* however its Hamming distance to some other *GF_y_* in the submatrix *A* can be > hamming_cutoff. Such features are removed from the submatrix thus, only features whose pairwise Hamming distance is <= hamming_cutoff remain in *A*. These remaining GFs are grouped into a new feature that has the same presence/absence pattern as the *GF_x_* with the lowest sum of Hamming distances to all other elements of *A*. This process is graphically explained in Fig S10. This grouping method is more intuitive than jaccard_filter and thus more suitable for most users. Other tools also implement feature clustering. Scoary [22] simply collapses all GFs with identical presence/absence patterns into one feature. Hogwash [14] method allows users to specify a key by which GFs will be grouped. The purpose of this grouping is to reduce the multiple testing burden. In practice, it is difficult to obtain such reliable keys as hierarchical databases like KEGG [135] are not large enough to assign function to many GFs in a pangenome. We could use Spearman or Pearson correlation with a defined cut-off to group GFs as used in PhenoLink [136]; however, Spearman correlation coefficient is used for measuring the monotonic relationship between two continuous or ordinal variables. On the other hand, Pearson correlation coefficient is a parametric measure of only continuous variables, and it is not suitable for correlating binary vectors. While it is technically correct to calculate Spearman's correlation coefficient for binary values, it may result in many ties and the interpretation is not as straightforward. We thus believe that the methods presented here are the most suitable for collapsing correlated features.

We applied hamming_filter to the *L. reuteri* dataset with hamming_cutoff = 3. The filter collapsed 1928 GFs into 495 new GFs. Many of these new GFs (50%) were a result of grouping just two GFs. A maximum of 32 GFs were grouped into one GF. The full distribution of collapsed GFs is depicted in Fig S11.


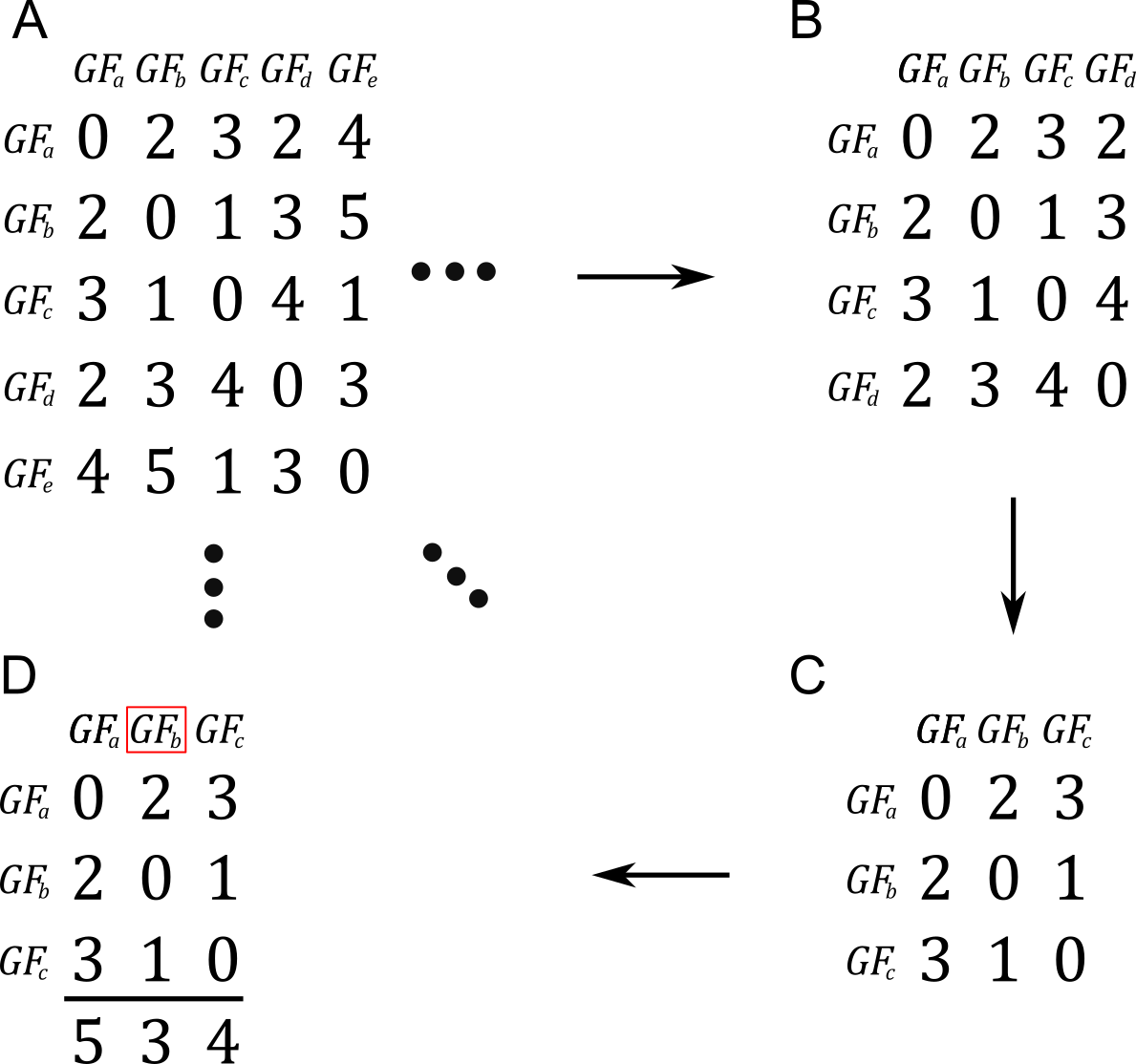


**Figure S10.** Description of the hamming_filter. First a Hamming distance between all *GF_a_*, *GF_b_* … *GF_n_* is calculated (A). Then *aurora* loops through all GFs. If *GF_a_* has a Hamming distance to any *GF_x_* lower or equal to hamming_cutoff (default 3) then the submatrix of such GFs is extracted (B). Additionally, only GFs that have a Hamming distance to all other GFs lower or equal to hamming_cutoff are retained (C). Next, the distance to all GFs is summed across all GFs, and the GF with the lowest sum (*GF_b_* in this case) is the representative GF for the whole cluster (D). In the next step *GF_a_*, *GF_b,_* and *GF_c_* would be removed, and the loop would continue.


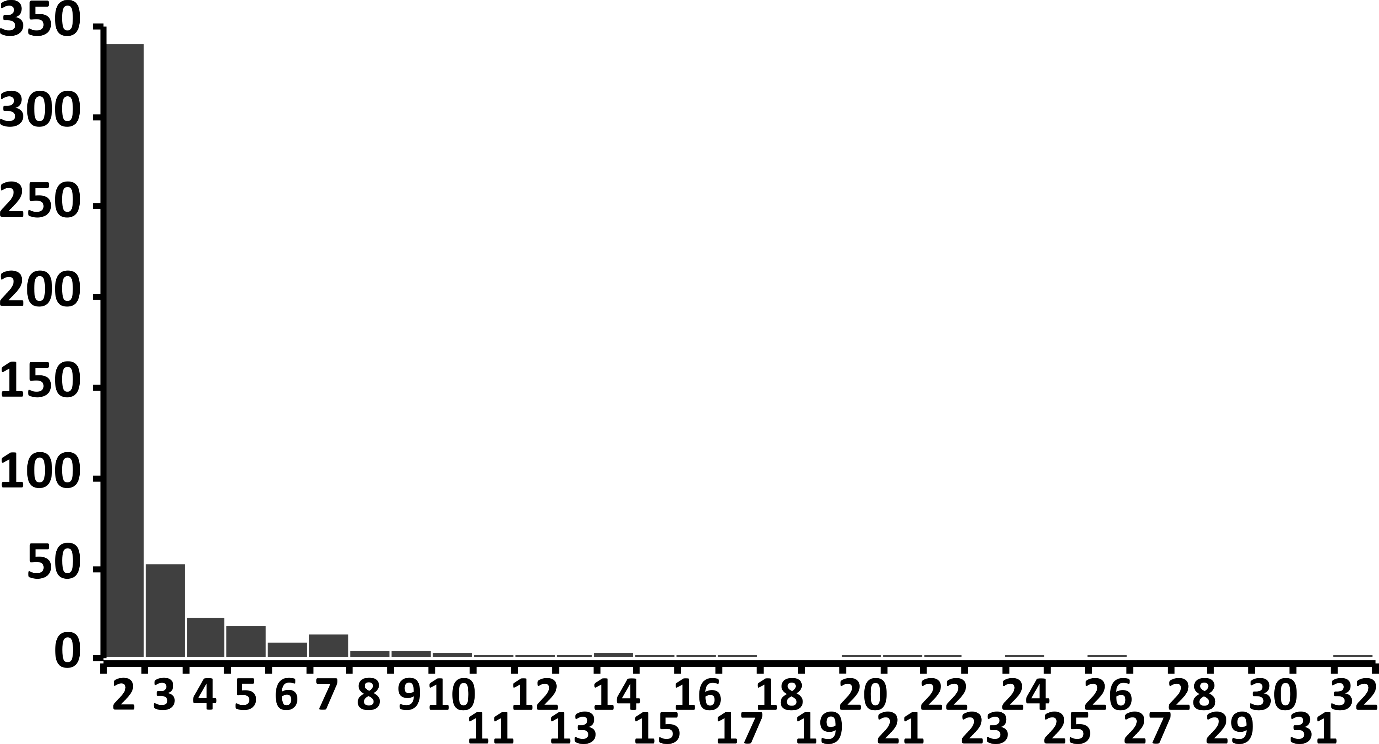


**Figure S11.** Histogram of GFs collapsed by hamming_filter with hamming_cutoff = 3. The histogram shows how many GFs (*x*-axis) were used to construct the 495 new GFs. The majority of the newly created GFs were constructed from only a few collapsed GFs.

## Removing outliers in a phylogenetic tree or distance matrix

Population adjustment methods are used to account for the relatedness among strains. Outliers can significantly distort population adjustments. For example, if the population adjustment technique uses principal components [21], outliers can cause the principal components to be dominated by the variation in the outlier observations, leading to a biased representation of the population structure. Applying linear models used by Pyseer [21], DBGWAS [10] and Bugwas [11] which rely on principal components could thus lead to spurious genotype-phenotype correlations and loss of power to detect significant associations.

Our package has an option controlled by the parameter reduce_outlier to account for the outliers in the phylogenetic reconstruction. This option does not use any strict threshold to implicitly remove the outliers. Instead, it aims to identify edges of phylogenetic tree or strains whose large phylogenetic distance could distort the population adjustment. If bagging parameter is set to phylogenetic_walk and the input is a phylogenetic tree, then the outlying branches of the tree are reduced. If the user supplied a phylogenetic distance matrix and selects phylogenetic_walk then the matrix is first converted to a tree using the Neighbor-Joining algorithm implemented in the ape package [132]. If random_walk and phylogenetic distance matrix are being used, then the pairwise distances are reduced. If the user supplies a phylogenetic tree and selects random_walk, then the tree is first converted to a distance matrix using cophenetic.phylo() function from the ape package [132]. After the initial pre-processing, either branches of the phylogenetic tree or pairwise distances of the distance matrix are collapsed into a single vector and z-score is calculated for each value. The distribution of z-scores of the *L.* *reuteri* phylogenetic tree is depicted in Fig S12. Values that are above a cutoff_outlier (default 3) are reduced to a value that is equal to cutoff_outlier multiplied by the standard deviation of the vector. Reducing the influence of outliers is especially recommended when phylogenetic_walk is used as a method for adjusting for the population structure. This is because – as shown below – phylogenetic_walk is more sensitive to outliers in the data than random_walk.


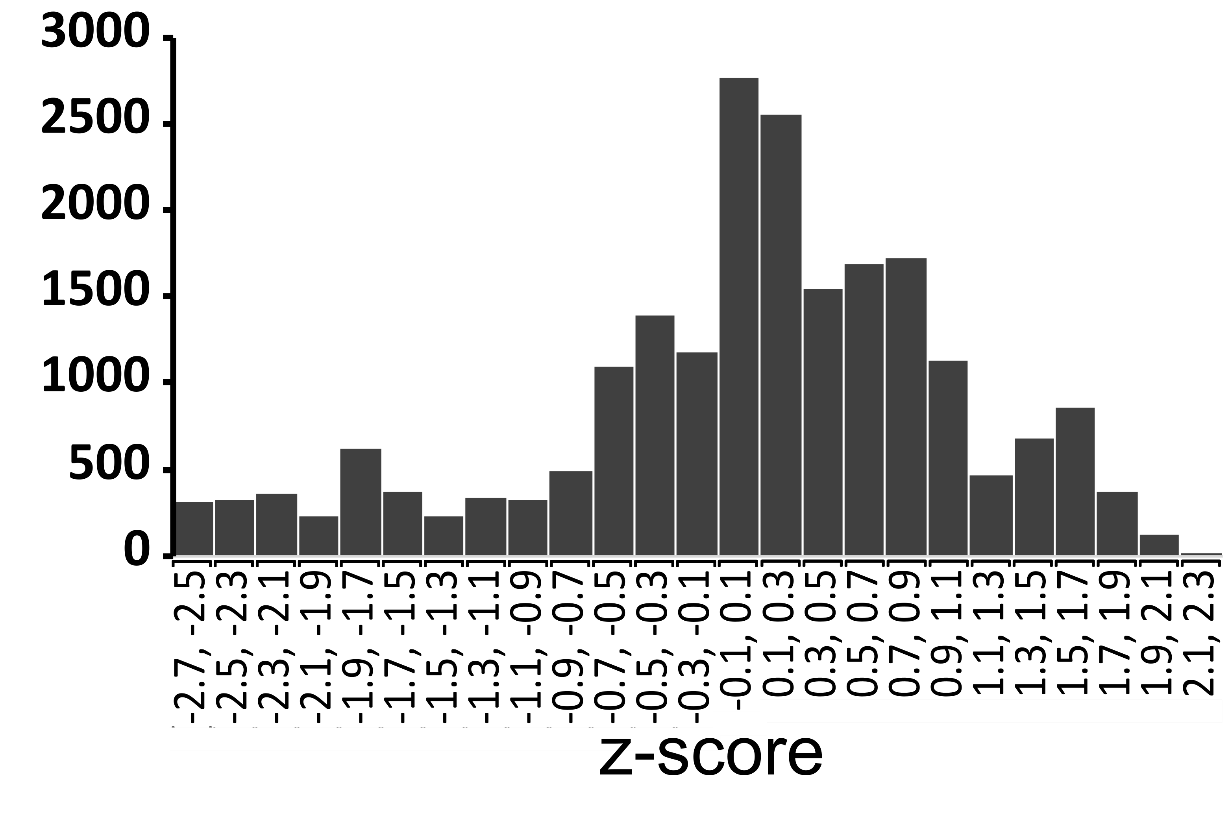


**Figure S12.** Histogram of z-scores calculated from branches of the phylogenetic tree of *L. reuteri*. Distances that are above cutoff_outlier (default 3) are reduced. In this case, no outliers were detected.

## Population adjustment methods in *aurora*

The *aurora* package implements two methods that resample the original dataset to produce a new sample which is adjusted for strain relatedness. In some cases, the original dataset is over-sampled (this means that the new dataset is larger than the original) in other cases it under-samples the dataset. Unlike previous approaches [1,2] our approach does not assume that the analyzed phenotype is not collinear with the phylogeny (see Fig 3. in the main text). We present two methods, random_walk and phylogenetic_walk. The random_walk sampling method does not explicitly capture the full population structure but captures only a trend (e.g., clonal lineages are sampled with lower probability). In the random_walk method, all strains tend to be sampled at least once and thus no variability is lost. Construction of phylogenetic trees is sensitive to model assumptions, and data quality, and may yield different topologies for the same set of strains. This can lead to potential confounding in downstream analyses that rely heavily on a single tree. Relying strictly on one phylogenetic representation may not be a good approach, especially in cases where the inferred topology is not well supported by bootstrap values. random_walk can be advantageous in three scenarios (i) the support bootstrap values are low or the phylogenetic distance matrix is not reliable (*i.e.,* the genome assemblies are incomplete or contaminated) (ii) the dataset potentially contains a large number of mislabelled strains – strict population adjustment would lead to the domination of mislabelled strains in the sample (iii) for large datasets (> 2000 strains) with multiple classes, random_walk might be a better option since it is ~100 times faster than phylogenetic_walk. On the other hand, phylogenetic_walk is an exact representation of the population structure. This method is preferable if there are a few potentially mislabelled strains and if the phylogenetic tree/matrix is of good quality. phylogenetic_walk is the default method.

Both bagging options require a set of parameters that constrain the sampling. The main parameter is bag_size which controls the number of strains that are selected per each class. The number of strains in each resampled class should be the same. If the user does not specify this argument, then the bag size is 5× the size of the smallest class. The maximum number of strain repetitions in a bag can be set by modifying the argument max_per_bag. By default, the argument is set to 0.2×bag_size. This ensures that none of the strains will represent more than 20% of the training dataset. In Threshold Calculation Phase the number of repetitions of the mislabelled strains is constrained. The upper constrain is calculated as:

$$max\_misslablel=\frac{bag\_size}{N}+\left\lfloor bag\_size\cdot0.1 \right\rfloor$$

Equation 3.

Where max_misslabel is the maximum number of repetitions any mislabelled strain is allowed to have in the training dataset. This constraint is in place to ensure that the mislabelled strains do not dominate the training dataset. *N* is the number of strains in a particular class and bag_size is the number of strains that will be sampled. It can happen that the mislabelled strain was not selected at all. In such a case, a random strain is removed from the bag and replaced with the mislabelled strain.

We commence by elucidating the random_walk population adjustment method. Let *N* be the number of all analyzed strains and *X_N×N_* be a phylogenetic distance matrix between all analyzed strains. In the first step a submatrix *Y_L×L_* is sliced from *X_N×N_* where *L* are strains belonging to a particular class. The first strain of the training dataset is selected randomly. Then, a row representing distances from the newly selected strain to other strains is sliced from *Y_L×L_*. This vector is used as a vector of probability weights in a randomized selection of the next strain. When the new strain is selected, a new row of probability weight is sliced again. The process continues until the desired number of strains is selected. During this process, strains that exceed either max_per_bag or max_misslabel are removed from *Y_L×L_*. The random_walk algorithm is run on every class separately and then the results are merged to create the final training dataset.

phylogenetic_walk works with a tree rather than a distance matrix. A number of steps are the same as in random_walk: bag size, max_per_bag, and max_misslabel are calculated the same way. The difference is in the selection of the training instances. The phylogenetic_walk selects a subtree *T* from the supplied phylogenetic tree that only contains strains of one class. Then the tree branch lengths are scaled to be between 1 and 1000 using min-max scaling. This ensures that all branch lengths are large positive numbers. Unlike in random_walk, each strain is selected independently of each other. First, two new subtrees *T_L_* and *T_U_* are extracted from *T*. *T_L_* is a tree that contains all nodes in the lower part of *T* (see Fig S13 for reference). Likewise, *T_U_* is a tree that contains all the nodes in the upper part of *T*. A probability weight is calculated for each subtree *T_L_* and *T_U_* as:

$$W_{x}=D_{x}+\sum_{i=1}^{n} L_{i,x}$$

Equation 4.

Where *W* is the probability weight, *x* is *U* or *L* for either the upper or lower part of *T*. *L_i_* is an *i-th* branch length of either *T_L_* or *T_U_*. *D* is the edge length of the two edges closest to the root of *T.* Once *T_L_* or *T_U_* is selected then they become *T* and the process is continued until a tree tip is reached. The process is visualized in Fig S13.


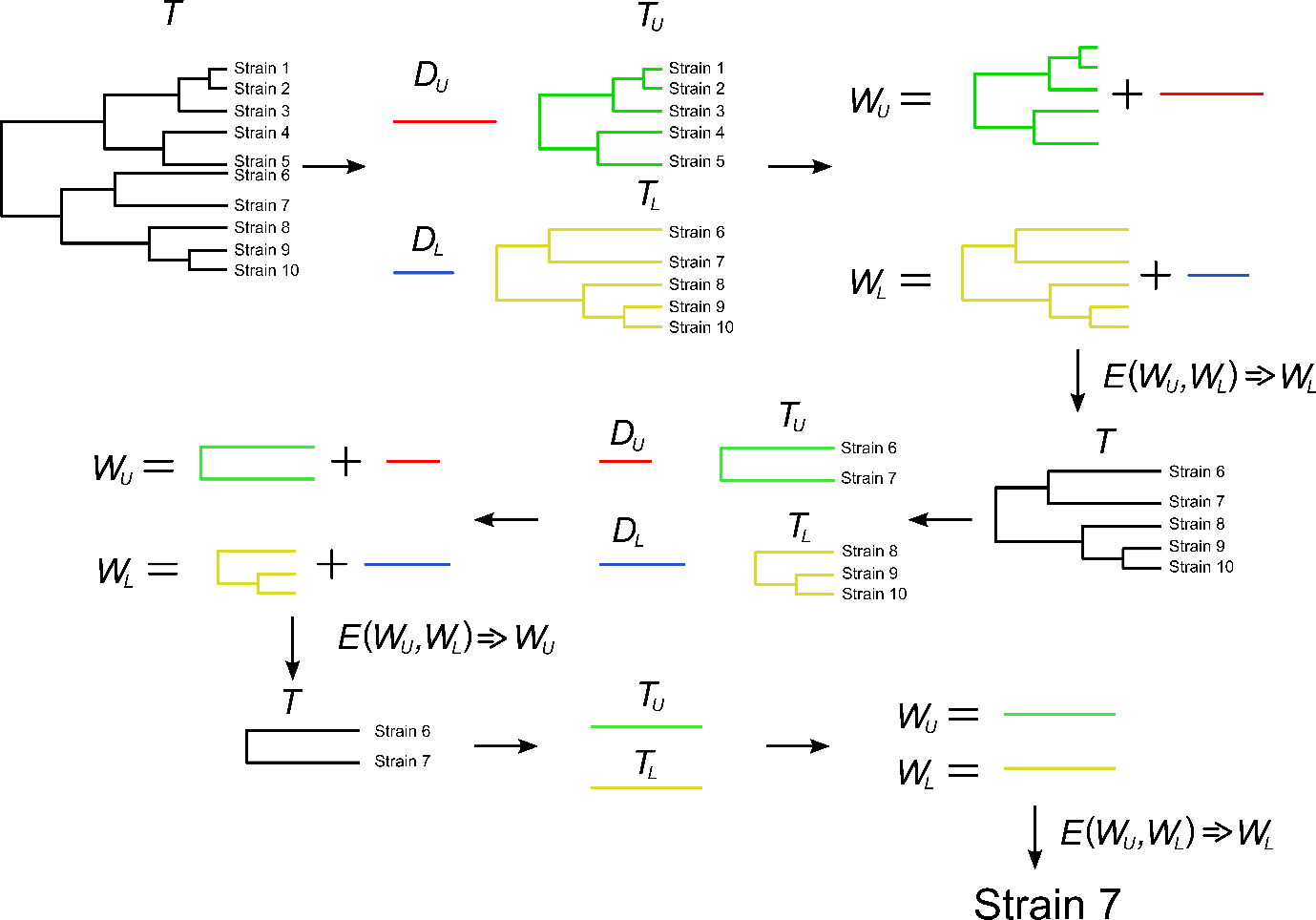


**Figure S13.** Visualized selection of one strain by phylogenetic_walk algorithm. First, a phylogenetic tree *T* is split into two subtrees *T_L_* and *T_U,_* and branches *D_L_* and *D_U_* that represent the distance from the root to the nearest two nodes. *W_L_* and *W_D_* are sums of branch lengths of subtrees *T_L_* and *T_U_* and they serve as probability weights used in a random selection of the next *T*. The process continues until a tip is reached. The whole process is run until a desired number of strains is reached (argument bag_size).

We ran both algorithms on simulated phylogenetic trees and the *L*. *reuteri* dataset (Fig S14 and S15 respectively). Three trees were simulated: (1) an equidistant tree where all branches have the same lengths (Fig S14AB); (2) A tree with an outlier in which the distance from the nearest node to the outlier is three times the length of the other branches (Fig S14CD). (3) A tree with a clonal lineage (Fig S14EF). The data were simulated with bag_size = 1000 and max_per_bag set to infinity. One hundred training datasets were simulated.

As expected, the equidistant tree showed that when the tree is regular, the analysis does not have to be adjusted for population structure because both methods function as random sampling. When there is an outlier, random_walk on average selected this strain only 78.28 times compared to phylogenetic_walk 98.17. Using the phylogenetic_walk method would lead to the domination of the outlier as it represented almost 10% of the whole training dataset.

We were also interested in how much a clonal expansion influences both methods. To this end, six clonal strains were added to one of the terminal branches. Five strains had an intra-cluster distance of 0.1 and one strain on the left side had a distance equal to 1 to the five clonal strains. The length of all other branches in the tree was 10. Since these strains are almost identical, they represent a repeating observation. By summing counts of all these clones, we should get an average count that other strains have. Using phylogenetic_walk, the sum of the average counts of the clonal lineage was 66.24, and the average count of all the other strains was 62.25. The method thus correctly represents the clonal lineage as just one strain. On the other hand, the sum of the average counts of the clonal strains in random_walk was 234.14 while the average count of other strains was only 51.76. Thus, clonal explosion influences only random_walk algorithm. This can be advantageous because each strain was sampled at least once for each bag and therefore no information was lost. It should be noted that the clonal expansion also influenced the sampling counts of related strains not just the clones (Fig S14E**)**.


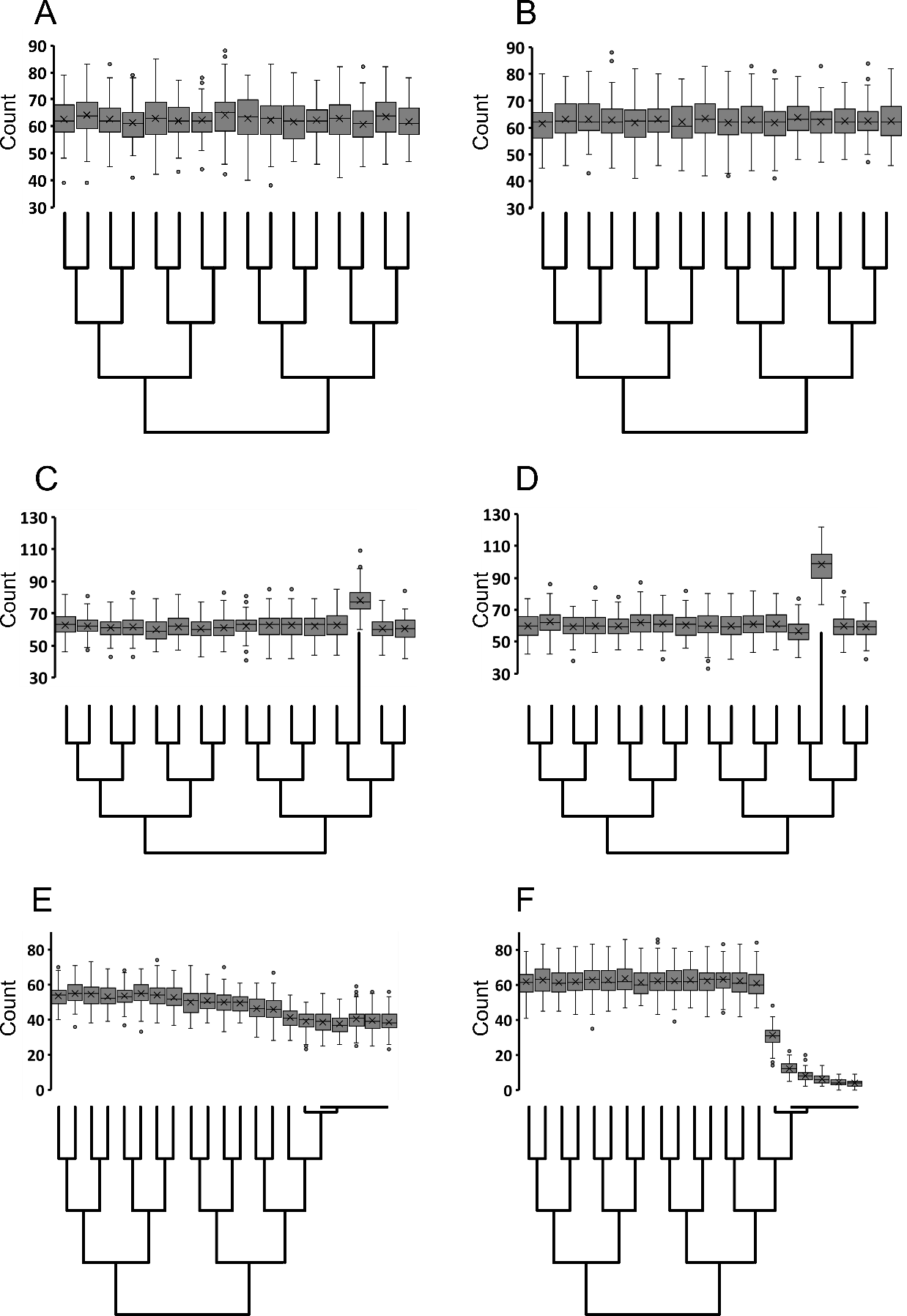


**Figure S14.** Testing properties of the random_walk and phylogenetic_walk algorithms on three phylogenetic trees. The *x*-axis shows the average number of times a strain was sampled into a bag. The size of the bag was 1000 and 100 bags were simulated. A and B show equidistant trees sampled with random_walk and phylogenetic_walk respectively. C and D show a tree with an outlier sampled with random_walk and phylogenetic_walk respectively, and E and F show a tree with a clonal explosion sampled with random_walk and phylogenetic_walk respectively.

Both methods were also applied to the *L. reuteri* dataset (Fig S15AB**)**. The trend is the same as shown using the simulated data. The random_walk algorithm samples the data more evenly and there are no large fluctuations in the sample counts. In contrast, the phylogenetic_walk algorithm prioritizes strains that are phylogenetically very distant from the rest of the population. phylogenetic_walk is thus an accurate representation of the phylogenetic relationships while random_walk discerns broader trends within the phylogeny which may not fully encompass the nuanced population structure of the species. In some cases, the best strategy seems to be to use random_walk in the aurora_pheno() function. After removing the mislabelled strains, the user can apply the more accurate phylogentic_walk in the function aurora_GWAS(). Both functions aurora_pheno() and aurora_GWAS() use phylogenetic_walk unless otherwise specified.


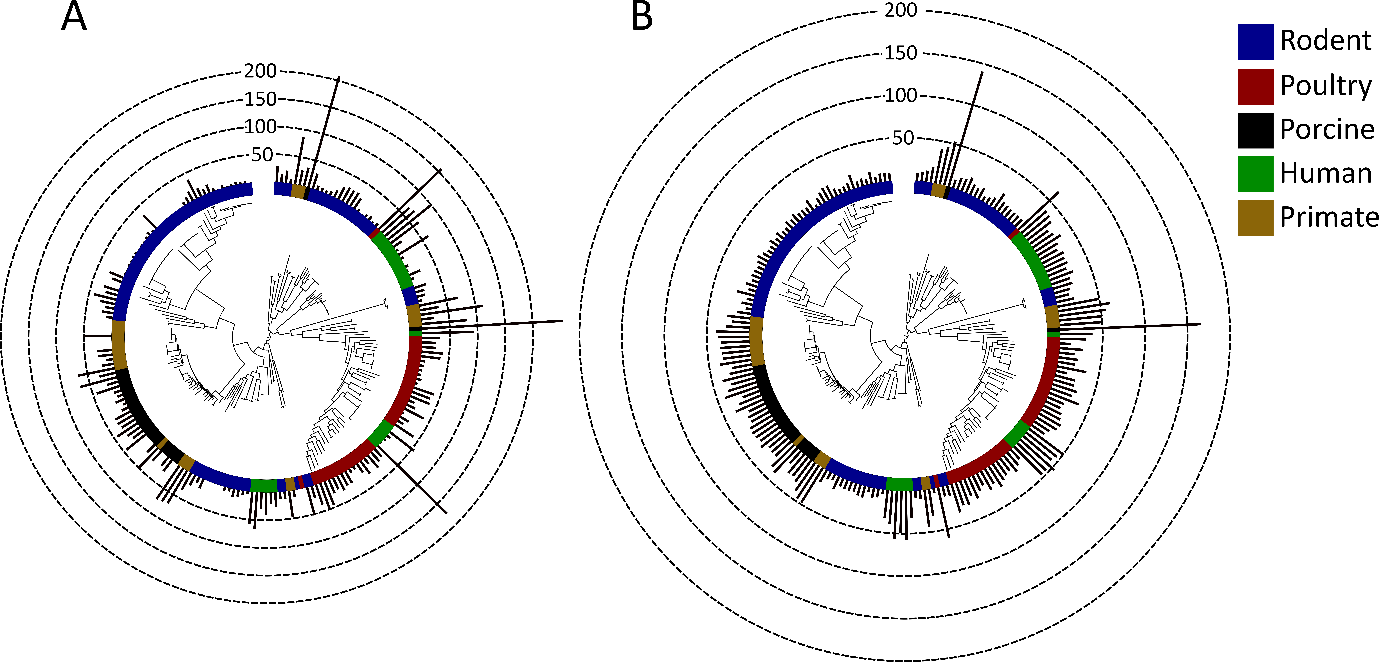


**Figure S15.** phylogenetic_walk (A) and random_walk (B) were used to resample *L. reuteri* dataset. Each class was resampled to reach 1000 strains (argument bag_size = 1000). The black columns show how many times was the strain repeated in the bootstrapped dataset. The construction of the tree is described in the methods part of the main text.

## The four machine learning methods and their hyperparameters

In the next phase, four machine learning algorithms are trained on the resampled datasets and then used on the original dataset to derive classification probabilities. aurora was designed to be an automated tool that should not require a lot of fine-tuning from the user. Thus, one of the main criteria for choosing a suitable ML algorithm was the number of hyperparameters. Additionally, we considered the interpretability of feature importances as another crucial parameter in algorithm selection. The ML classifiers also need to be able to produce classification probabilities. Additionally, we wanted the selected algorithms to belong to different categories of classification machine learning algorithms. Consequently, we conducted experiments using various ML algorithms on the *L. reuteri* dataset (Fig S16), namely Random Forest (multiclass, one vs rest and one vs one configuration), AdaBoost (multiclass, one vs rest and one vs one configuration) Classification And Regression Trees – CART (multiclass, one vs rest and one vs one configuration) logistic regression (multiclass and one vs one configuration) and multiclass Linear Discriminant Analysis. To test these ML methods we mislabelled one randomly selected rodent strain into each class and built 100 training datasets with bag_size = 100 for each class. The trained models were then used to classify all rodent strains and the mislabelled strains.

It is known that *L. reuteri* is well adapted to survive in the GIT of rodents [64,66,87,127]. Therefore, if one rodent isolate is mislabelled into another class, this strain should still retain a high classification probability in the rodent class. It is not desirable for the classifiers to have a high variance. In such a case, mislabelled strains would have a high classification probability in their new classes, and it would not be possible to identify allochthonous strains. Out of all the tested ML methods, multiclass Random Forest, and CART with logistic regression in all configurations had shown that they are still able to recognize rodent strains even after they were intentionally mislabelled. Thus, multiclass Random Forest, one vs one CART, and one vs rest logistic regression were implemented as classification algorithms into *aurora*. Note that all ML algorithms selected use different configurations. Additionally, all the selected ML methods work on a different basis; Random Forest is an ensemble method, logistic regression is a generalized linear model, and CART is an individual learner. We also decided to implement multiclass AdaBoost. While the performance on the *L. reuteri* dataset was poor this dataset might be too complex for this model. Other simpler datasets may benefit from the simplicity of weak learners (stumps in this case) and a mechanism for early stopping. As such AdaBoost is not as easy to overfit as all the other ML algorithms. AdaBoost – as well as other ML algorithms – can be disabled by setting adaboost = FALSE in aurora_pheno().


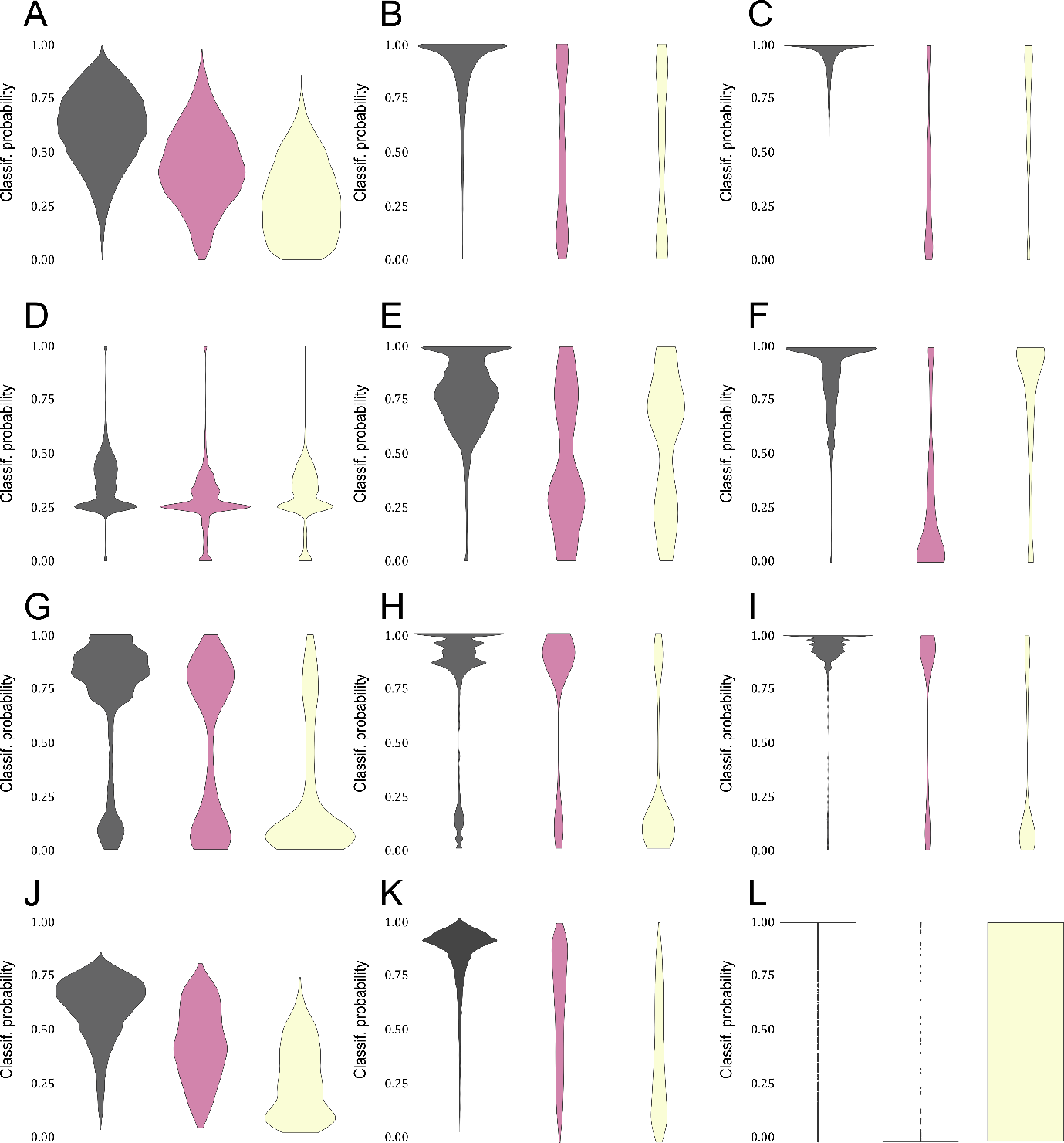


**Figure S16.** Testing of multiple machine learning methods on *L.* *reuteri* dataset. Gray color – rodent isolates that have not been mislabelled. The plotted values of this set are classification probabilities in the rodent class. Pink color – rodent isolates that have been mislabelled to any other class. The plotted values of this set are classification probabilities in the rodent class. Yellow color – these are also rodent isolates mislabelled into a different class, but the plotted values are classification probabilities in the class to which they were mislabelled. A) multiclass Random Forest B) one vs rest Random Forest C) one vs one Random Forest D) multiclass AdaBoost E) one vs rest AdaBoost F) one vs one AdaBoost G) multiclass CART H) one vs rest CART I) one vs one CART J) one vs rest logistic regression K) one vs one log regression L) multiclass Linear Discriminant Analysis.

Log regression was regularised with *l1* penalty and the liblinear solver was used in all cases. The regularization term *C* was set to 0.5 in these simulations, and it is also a default in *aurora*. The user can change this by modifying the parameter C_val. Log regression is run using a Python 3.0 and scikit-learn library (<https://scikit-learn.org/stable/>). CART does not require any hyperparameters. Package rpart (<https://cran.r-project.org/web/packages/rpart/>) is used to construct the trees. First, a tree is constructed using a complexity parameter cp = 0.01, minsplit parameter set to 2 and minbucket parameter set to max_misslabel + 1. These parameters result in overfitted trees. Four-fold cross-validation is carried out and cross-validation error is calculated for each node. Then a threshold is calculated as one standard deviation of the smallest cross-validation error plus the smallest cross-validation error. The cp cutoff corresponds to the largest cross-validation error that is still below the threshold. All nodes whose cp is lower than the new cp are pruned. This new tree is then used to classify all analyzed strains.

If Random Forest and AdaBoost are used, users can either supply their hyperparameters or set argument fit_parameters = TRUE. Finding hyperparameters for Random Forest and AdaBoost algorithms entails a more intricate computational process than in the previous two cases, necessitating a grid search. Random Forest is run using an implementation in R package randomForest (<https://cran.r-project.org/web/packages/randomForest/index.html>) and AdaBoost is run with scikit-learn library (<https://scikit-learn.org/stable/>). If random_forest and fit_parameters are both set to TRUE then a grid search is run over values shown in Table S1. Due to extensive resampling, and thus multiple strain repetitions in the dataset, the split ratio is 30% (training) to 70% (testing). The grid search has to be run with multiple datasets because both resampling methods (random_walk and phylogenetic_walk) are stochastic. The number of repeating grid searches is controlled by the parameter repeats (default: 10). The combination of hyperparameters that yield the highest median accuracy is selected for all further models. Fitting a hyperparameter into AdaBoost is analogous (grid search parameters are in Table S1). SAMME.R algorithm is used in all cases. *aurora* uses only stumps as weak learners in AdaBoost as higher tree depts resulted in overfitting. Fitting of hyperparameters can take a few minutes to hours, with the runtime largely dependent on the value of parameter repeats.

**Table S1**. Hyperparameter space used in grid search for Random Forest and AdaBoost algorithms.

| Random Forest | | |
| --- | --- | --- |
| Parameter | Values | Explanation |
| sampsize | 0.2, 0.4, 0.6, 0.8 | Determines the size of the random sample used to build each tree. |
| mtry | 10, 50, 200, 500, 1000, 2000 | Controls the number of features considered at each split. |
| ntree | 10, 50, 100, 500, 1000 | Determines the number of trees in the forest. |
| maxnodes | 4, 8, 12 | Limits the maximum number of terminal nodes in each tree. |
| AdaBoost | | |
| n_estimators | 10, 50, 100, 500 | Determines the number of weak learners to combine. |
| learning_rate | 0.01, 0.1, 0.5, 1, 1.5 | Controls the contribution of each weak learner in the final strong learner. |

## Threshold Calculation Phase

Once the features are filtered and grouped and the best hyperparameters are fitted, *aurora* is ready to enter the next stage. The purpose of this stage is to find out if the entire species is adapted to the analyzed phenotype and to derive multiple threshold distributions that are used to identify the mislabeled strains in a later stage. This stage is named the Threshold Calculation Phase. The process is similar for all ML algorithms. First, one strain is randomly selected from one class and then incorrectly labeled as belonging to a different class. This mislabeling step is repeated for all the classes in the dataset. Thus, if the user analyses a phenotype with four classes three strains in each class are selected and mislabeled into the remaining three classes. Then this partially mislabeled dataset is resampled using either random_walk or phylogenetic_walk. The ML classifiers are trained on this data and then used to predict the classification probabilities of the original dataset. Classification probabilities of all classes are recorded. Because both resampling algorithms and the mislabeling are stochastic, this process has to be repeated multiple times. The number of repetitions is set by the argument no_rounds (default 100). The value of this argument along with the number of strains, features, and analyzed classes are the main factors influencing the runtime of aurora_pheno(). The users can opt to lower no_rounds but this should be done with caution because the power needed to identify both autochthonous and allochthonous strains may be lost.

Next, *aurora* calculates if the intentionally mislabeled strains have significantly lower classification probabilities in the new class than non-mislabeled strains in the same class. If the difference is significant, then the two classes are separable, and thus the species is adapted to both classes. A two-sample Kolmogorov-Smirnov test is used to calculate the *p*-value. The choice of the Kolmogorov-Smirnov test over a more common two-sample Mann-Whitney U test is justified here. Both tests are non-parametric and compare two independent distributions, but the Kolmogorov-Smirnov test is sensitive to differences in both the location and shape of the distributions, while the Mann-Whitney U test is primarily sensitive to differences in location (*i.e.,* median). It is important to take into account the shape of the distribution because there may be multiple mislabeled strains (*i.e.* mislabeled in the original data not intentionally by *aurora*) whose classification probability should be high in the new class. Additionally, the Kolmogorov-Smirnov test becomes more powerful as the size of the sample increases. The test is calculated twice per pair of classes. First, it is tested if strains mislabeled from class A to class B have lower class B classification probabilities than non-mislabeled class B strains and vice versa with strains mislabeled from class B to class A. If both *p*-values are above 0.05 the two classes should be considered indistinguishable hence without specific adaptation.

We ran 100 iterations with all four ML algorithms. The matrices with the *p*-values are shown in Fig S1. All ML algorithms predicted that *L*. *reuteri* is adapted to all hosts. These results agree with already published conclusions where it was shown that *L. reuteri* forms deep-branching phylogenetic rodent and poultry clusters which point to the fact that these strains are adapted to their host [16]. On the other hand, phylogenetic clusters containing primarily human, porcine, and primate strains originated more recently [16]. Additionally, it was shown that two human *L*. *reuteri* isolates were surprisingly not able to stably colonize humans [64]. This uncertainty regarding the colonization ability of human isolates is reflected in the *p*-value matrices (Fig S1). Random Forest, and to some extent AdaBoost, log regression, and CART all show that if human isolates were misplaced into a new class, then its classification probabilities in the new classes are high. This indicates that a proportion of human isolates are not restricted only to human hosts. While human isolates are certainly more diverse than other groups of isolates, they are not significantly similar to any other class and thus represent a unique class. Taken together, we have shown that *aurora* correctly classified *L. reuteri* as a host-adapted species. The ML algorithms showed that isolates from all habitats are mutually distinguishable and thus acquired specific adaptation genes. It was also shown that using multiple classification ML algorithms is desirable because the results can differ. The ML algorithms will complement each other which is especially useful for the analysis of complex phenotypes.

## Outlier Calculation Phase

Even if none of the analyzed classes are distinguishable, *aurora* continues to the next step which is Outlier Calculation Phase. In this step, *aurora* obtains a set of phenotype classification probabilities for each strain and compares them to sets of probabilities calculated in the previous step. The purpose of this is to find out if the classification probabilities of a particular strain resemble the classification probabilities of non-mislabeled or mislabeled strains and thus identify the true phenotypic class of each strain.

The process is similar to that in the Threshold Calculation Phase but none of the strains are intentionally mislabeled. First, the original dataset is resampled by either random_walk or phylogenetic_walk and the resulting dataset is used for training the ML models. The models are then used to classify the original dataset. This process is repeated multiple times (specified by argument no_rounds). In this phase, several other values are recorded. Area under (AUC) a receiver operating characteristic curve (ROC) is recorded for each round. The sets of AUC values for each ML algorithm are part of the output. The user should inspect this set to verify that none of these values are noticeably lower or higher than the rest. The AUC values for each ML algorithm are in Fig S17 and Table S2. The AUC values are close or equal to 1 in almost all cases which shows that the models are likely overfitted. It has already been shown that models can easily learn non-causal features from a pangenome matrix, even if cross-validation is used [47,137]. The models that *aurora* constructed have high variance, but this is not an obstacle for identifying mislabeled strains since *aurora* does not rely on classification probabilities calculated in this step but uses threshold distributions calculated in the Threshold Calculation Phase.


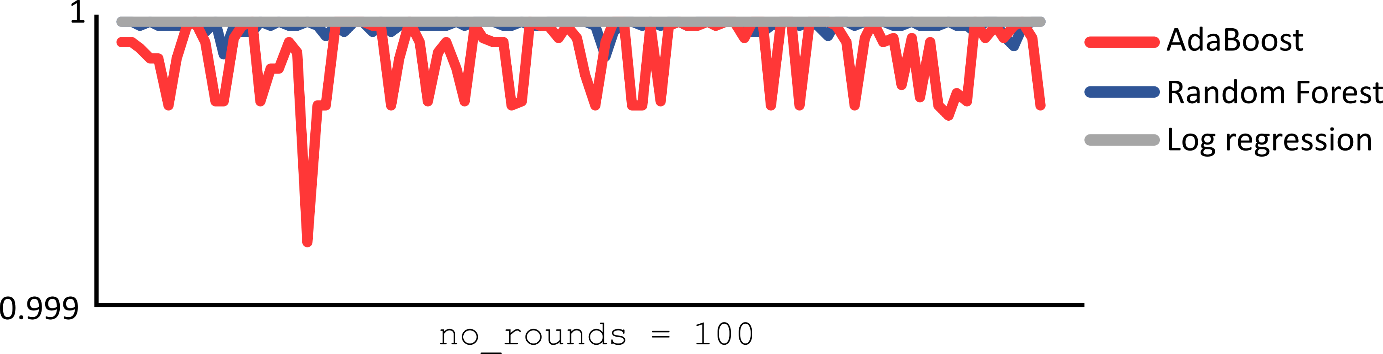


**Figure S17.** AUC values of three ML models calculated in the Outlier Calculation Phase. The values were calculated based on *aurora* run with the *L. reuteri* dataset.

The feature importances are recorded each round for each tool. In case of Random Forest, the importance that is recorded is the “mean decrease of Gini” and the “mean decrease of accuracy”. For AdaBoost, it is the mean decrease of Gini. For log regression it is the coefficients *β_x_* that are associated with each predictor *X* and for CART it is the sum of the goodness of split measures for each split in the tree. The CART feature importance does not only consider the primary feature (the feature used in the tree) but also all surrogate features. For more information about the feature importances in CART, see the rpart vignette (<https://cran.r-project.org/web/packages/rpart/vignettes/>). The CART feature importances are then scaled to sum to 100.

Next, the importances of each feature are extracted, values that are equal to zero are removed and a median of the remaining values is calculated. In addition, to the feature importances, *aurora* output also contains the number of strains where the feature is present/absent. Examining the feature importances can be useful but it should be noted that the value of importance is not directly proportional to the probability under the null hypothesis – *p*-value [138]. A particular feature can have high importance (especially Gini or accuracy) while only present in a few strains. In the case of models that assume linear relationships (*i.e.,* log regression) the feature importance is proportional to the *p*-value while non-linear algorithms can have a bias towards features present in multiple classes. Instead of relying on feature importances *aurora* uses the function aurora_GWAS()to find interpretable and accurate genotype-phenotype associations.

Function aurora_pheno() outputs values that are specific for each ML algorithm such as a distance matrix based on proximities calculated in Random Forest. The proximities represent the similarity between strains based on how often they appear in the same terminal node across all the trees in the forest. Such a matrix is generated from every constructed Random Forest classifier. Then both the cumulative sum and media of elements in the same row and column across all matrices are calculated. These two matrices are clustered and plotted (Fig S18) automatically if the user sets plot_random_forest to TRUE which is the default behavior. Examining these matrices can identify cases where two different adaptations to a habitat have evolved. It is also possible to identify other allochthonous strains which aurora_pheno() autochthonous but are clustered with other allochthonous strains. Before removing any additional strains, the user should keep in mind that this is a result of only one ML tool.


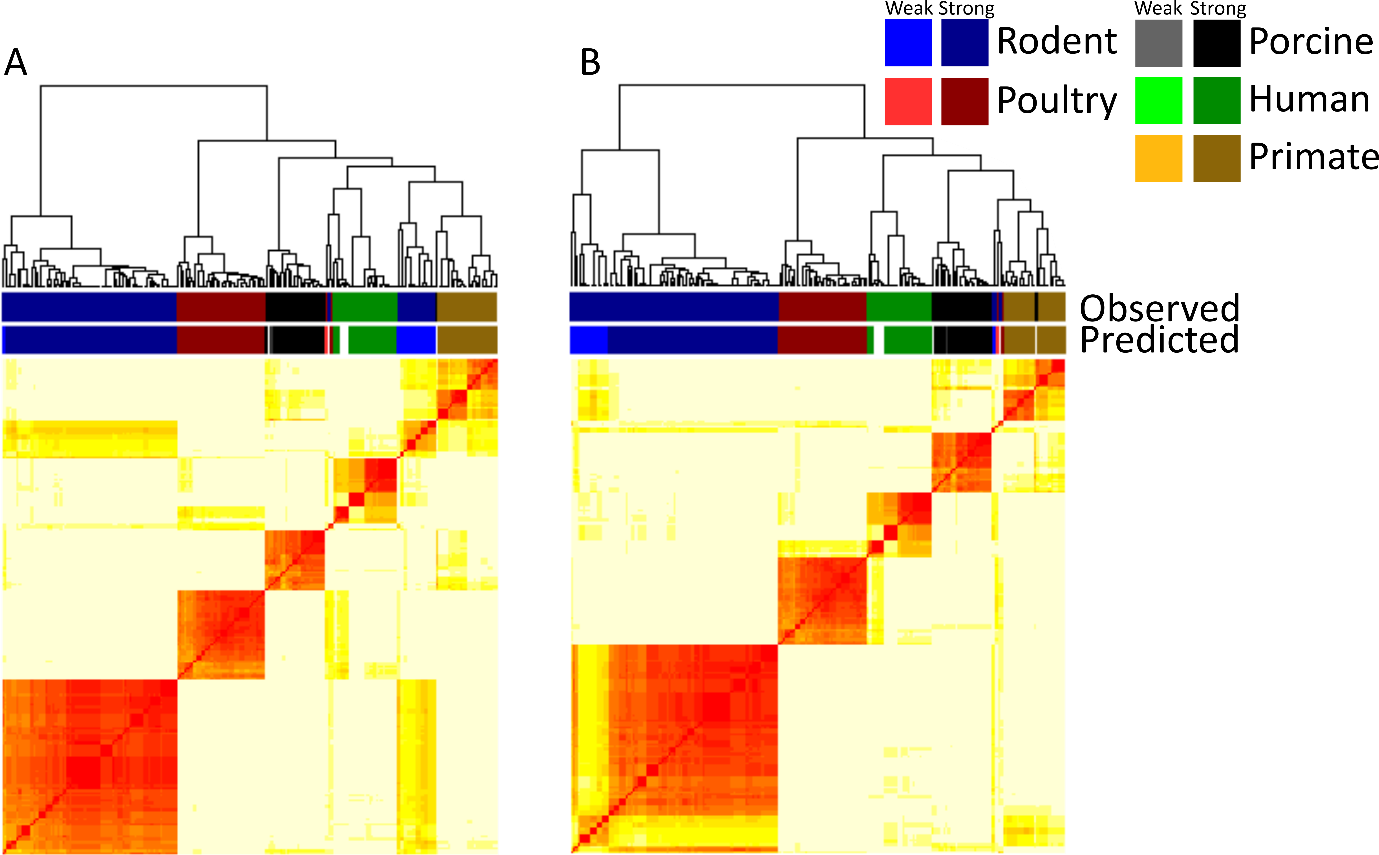


**Figure S18.** Distance matrices were calculated based on Random Forest proximities obtained in aurora_pheno(). The distance matrices were clustered with hierarchical clustering. The two color strips show the observed and predicted hosts. A) Distance matrix containing the sum of elements across the whole set of matrices obtained in aurora_pheno(). The matrix is the same as in Fig 6 of the main text. B) Distance matrix containing median calculated from all elements across the whole set of matrices obtained in aurora_pheno().

*aurora* calculates an additional matrix based on distances derived from CART models. The distance measure does not consider only the leaf nodes of a decision tree but takes into account the topology of the tree. The calculation of the distance measure is described in Fig S5. If CART is used, then three additional values are generated, the minimum, maximum, and most common number of splits of the computed CART models. This shows how many features are needed to separate each pair of habitats. The values for *L. reuteri* are shown in Table S2. The user can thus see that there is one feature that is present in all poultry strains and absent in all primate strains (or vice versa). These two particular classes are easy to separate while poultry and rodent strains or poultry and human strains require more splits.

**Table S2**. Results of *aurora* with CART model run on the *L. reuteri* dataset.

| Classes | Median of AUC | Modus of splits | Minimum splits | Maximum splits |
| --- | --- | --- | --- | --- |
| poultry vs rodent | 0.98 | 3 | 3 | 5 |
| poultry vs human | 0.97 | 3 | 2 | 4 |
| poultry vs porcine | 0.98 | 2 | 1 | 2 |
| poultry vs primate | 1 | 1 | 1 | 1 |
| rodent vs human | 0.97 | 2 | 2 | 2 |
| rodent vs porcine | 0.99 | 2 | 1 | 2 |
| rodent vs primate | 1 | 2 | 2 | 2 |
| human vs porcine | 1 | 1 | 1 | 1 |
| human vs primate | 1 | 2 | 1 | 2 |
| porcine vs primate | 0.99 | 3 | 2 | 5 |

## Finding allochthonous strains

The result of the Outlier Calculation Phase are sets of classification probabilities of each strain in each phenotypic class. In this step, these sets are compared to equivalent sets calculated in the Threshold Calculation Phase. An example of rules that are responsible for class prediction is shown in Equation 5. In this example, we consider three habitats of the analyzed species (habitats *A*, *B,* and *C*). There are four categories into which a strain can be classified. The categories are not mutually exclusive hence one strain can belong to multiple categories. If all terms *P, Q*, *R*, *S*, and *T* evaluated in Equation 5 are true then the strain is autochthonous in the habitat where it was isolated. If *P* in Equation 5 is false then Equation 6 is true, thus the strain is autochthonous in the isolation habitat, but it is not a typical genetic representative of the habitat population. These strains have a lower classification probability in the habitat in which they were isolated but at the same time, their classification probability in other habitats is not high enough to be classified as allochthonous. These strains have some important autochthonous markers, but some are missing. In practice, the absence can be genuine or a result of incorrect pangenome prediction or incompleteness of the assembly. If either *Q*, *R*, *S*, or *T* in Equation 5 are false then the strain might be autochthonous in a different habitat where it was isolated.

$$\forall i\left( KS\left( A,A_{i} \right)<\alpha\to\neg P \right), \forall i(KS(A,A_{i})\geq\alpha\to P)$$

$$\forall i(KS(A_{i},A_{B})<\alpha\to Q)\wedge\forall i(KS(A_{i},A_{B})\geq\alpha\to\neg Q)$$

$$\forall i(KS(B_{A},B_{Ai})<\alpha\to R)\wedge\forall i(KS(B_{A},B_{Ai})\geq\alpha\to\neg R)$$

$$\forall i(KS(A_{i},A_{C})<\alpha\to S)\wedge\forall i(KS(A_{i},A_{C})\geq\alpha\to\neg S)$$

$$\forall i(KS(C_{A},C_{Ai})<\alpha\to T)\wedge\forall i(KS(C_{A},C_{Ai})\geq\alpha\to\neg T)$$

Equation 5

Where *KS(X,Y)* is a function that uses the Kolmogorov-Smirnov test to calculate if a set *Y* is lower than set X. The function outputs the *p*-value of the test. *A* is a set of class A classification probabilities calculated in the threshold calculation phase. These strains were not mislabeled and belong to class A. *A_i_* is a set of class A classification probabilities of strain *i* calculated in the outlier calculation phase. Strain *i* in this example belongs to class A. *A_B_* is a set of class A classification probabilities calculated in the threshold calculation phase. These strains were mislabeled from class B into class A. *B_A_* is a set of class B classification probabilities calculated in the threshold calculation phase. These strains were mislabeled from class B into class A. *B_Ai_* is a set of class B classification probabilities of strain *i* (which belongs to class A) calculated in the outlier calculation phase. *A_C_* is a set of class A classification probabilities calculated in the threshold calculation phase. These strains were mislabeled from class C into class A. *C_A_* is a set of class C classification probabilities calculated in the threshold calculation phase. These strains were mislabeled from class C into class A. *C_Ai_* is a set of class C classification probabilities of strain *i* calculated in the outlier calculation phase. In this process, *aurora* uses only α = 0.05. Equation 5 shows only an example with three classes, but this process can be extended to an arbitrary number of classes.

*aurora* can also predict if a strain is a typical representative of the class (*e.g.,* strong, or weak colonizer – see Fig S18).

$$\neg P\wedge Q\wedge R\wedge S\wedge T$$

Equation 6

Where statements *P, Q, R, S, and T* were evaluated in Equation 5. If the above statement is true then the strain is autochthonous in the habitat where it was isolated, but it is not a typical strain. In such a case, the strain has low classification probabilities in the observed class (isolation habitat) however the strain has also lower classification probabilities in other classes. These strains should not be considered mislabeled. In fact, it is likely that these cases will be present even in datasets where the species is well adapted to the isolation habitat.

$$\left( \neg Q\wedge\neg R \right)\vee\left( \neg S\wedge\neg T \right)$$

Equation 7

Where statements *Q, R, S,* and *T* were evaluated in Equation 5. If the above statement is true, then the strain is autochthonous in habitats B and/or C. More specifically, if $(\neg Q\wedge\neg R)$ is true, then the strain is autochthonous in habitat B, and if the $(\neg S\wedge\neg T)$ is true then the strain is autochthonous in habitat C. If at the same time the term *P* is false then the strain was mislabeled in the original dataset.

$$\left( \neg Q\wedge R \right)\vee\left( Q\wedge\neg R \right)\vee\left( \neg S\wedge T \right)\vee\left( S\wedge\neg T \right)$$

Equation 8

Where statements *Q, R, S,* and *T* were evaluated in Equation 5. If the above statement is true, then the strain's true habitat is inconclusive. *aurora* thus cannot assign a definite classification label to the strain.

$$P\wedge(\neg Q\wedge\neg R)\wedge(\neg S\wedge T)$$

Equation 9

It is common for one strain to have multiple classification labels. Equation 9 shows an example of a strain observed in class A that is autochthonous in class A and class B while being inconclusive because of ambiguous probabilities in class C. The results of this step with *L. reuteri* and *Lactiplantibacillus plantarum* datasets are shown in Fig 6C in the main text.

## GWAS analysis

The purpose of the steps so far was to identify mislabeled strains. In the next step, these strains are removed and a GWAS analysis is conducted with the new dataset. This is carried out by a separate function aurora_GWAS(). This function can be run even when aurora_pheno() was not previously executed. In such a case, no strain will be removed. aurora_GWAS() requires a data frame with unique strain indices in the first column and the isolation habitat or any other phenotype in the second column. Additionally, the function also requires a phylogenetic tree or a distance matrix. These objects are the same as those required for aurora_pheno() however, aurora_GWAS() can also take a list produced by aurora_pheno(). Just like aurora_pheno(), aurora_GWAS() can also reduce the distance of outlying strains to the rest of the population. This optional step is again controlled by the argument reduce_outlier (default TRUE, cutoff_outlier = 3).

The user does not have to use results from all the ML algorithms. This is advised especially in cases where an ML algorithm produced high *p*-values in the Threshold Calculation Phase or if the ML algorithm shows very different results than all the other algorithms. CART for example is a simple model that can easily underfit while log regression can overfit the data. Any ML algorithm can be switched off by setting use_rf, use_ada, use_log, or use_CART to FALSE. By default, all ML algorithms are turned on.

If results from aurora_pheno() are provided then the user can also choose between two modes of consensus and strict. Consensus mode will remove only strains that were identified as mislabeled by all used ML algorithms. On the other hand, the strict mode will remove all strains that were identified as mislabeled by at least one ML model. Additionally, the user can also set the parameter rm_non_typical to TRUE (default: FALSE). This will lead to strains that were labeled as non-typical (weak colonizers) being treated as mislabeled strains. aurora_GWAS() produces a data frame that shows which strains were removed. This data frame can be used to map the result to a phylogenetic tree using iTOL [126].

The GWAS analysis starts with calculating a simple ratio $\frac{\alpha_{i}}{N_{i}}$ for each feature and each class where *α_i_* is the number of strains in class *i* where the feature is present, and *N_i_* is the number of all strains in class *i*. These values are part of the output.

Next, the dataset is resampled with either phylogenetic_walk (default) or random_walk. In this step, the best possible control of the population structure is necessary, and it is therefore recommended to use phylogenetic_walk. random_walk should be used only in cases where the phylogenetic reconstruction is not reliable or if the dataset contains distant outliers. By default, argument max_per_bag is set to 100000 (a number that cannot be reached) and bag_size to 1000. The latter value should be increased if the analyzed dataset has over ~300 strains per class.

After the dataset is resampled with a population-aware algorithm precision (Equation 10), recall (Equation 11), and F1 values (Equation 12) are calculated for each feature and each class. These values ​​are calculated so that each feature represents a node of a decision stump that divides the strains into two leaf nodes, those that do not have the feature and those that do. Precision, recall, and F1 are then calculated for this simple stump. Causal features will have the highest F1 scores.

$$P=\frac{{TP}_{ij}}{{TP}_{ij}+{FP}_{ij}}$$

Equation 10

$$R=\frac{{TP}_{ij}}{{TP}_{ij}+{FN}_{ij}}$$

Equation 11

$$F1=2\cdot\frac{P\cdot R}{P+R}$$

Equation 12

Where *TP_ij_* is the number of true positives. These are strains that have the feature *i* and belong to class *j*. *FP_ij_* is the number of false positives, *FN_ij_* is the number of false negatives and *P* and *R* are precision and recall respectively. F1 scores prove highly effective for identifying variants with high effect size and high presence ratio (see Fig S6). Therefore no previously proposed frequency cutoff is required [9]. While F1 values excel in detecting variants with a high effect size and high presence ratio, they are not particularly effective in identifying causal variants with low effect size and low frequency. To address this limitation, the function aurora_GWAS() employs the calculation of standardized residuals (Equation 13). Unlike F1 values, the standardized residuals show a more modest increase with rising presence ratios (Fig S6), making them well-suited for the identification of variants with low presence ratio and at the same time low effect size. Refer to the main text for a discussion on when to utilize F1 values and when to employ standardized residuals as a metric for identifying causal features. Running the function aurora_GWAS() takes only a few minutes.

$${SR}_{ij}=\frac{O_{ij}+E_{ij}}{\sqrt{E_{ij}}}$$

Equation 13

Where *SR_ij_* is a standardized residual of feature *i* in class *j*. *O_ij_* is the observed frequency of the feature *i* in class *j* and *E_ij_* is the expected frequency of the feature *i* in class *j*.

## Results of different machine learning algorithms on the *L. reuteri* dataset

The random forest model in *aurora* classified *L. reuteri* as a host-adapted species as evidenced by *p*-value matrices calculated in the Threshold Calculation Phase shown in Fig S1. Additionally, the distance matrix showing proximities calculated from the random forest model shows that most rodent strains tend to be classified together in one leaf node (Fig 6A in the main text). Moreover, defined clusters are obvious in the distance matrix and the clusters correspond to the isolation source. However, there is a cluster of weakly autochthonous rodent isolates that is further from the rest of the rodent strains. Surprisingly, these strains do not lack any of the above-described adaptation mechanisms, but their genomes contain a glutamate-dependent acid resistance regulator (group_2077), sensor histidine kinase AgrC (group_4159), surface protein G (group_2603) and a surfactin family lipopeptide synthetase A (group_2399). These genes are the best predictors for primate hosts as shown by *aurora*. Multiple primate isolates also possess genes that are important for rodent colonization. For example, 10 out of 25 primate isolates contain the full *ure* operon and 12 genomes contain a gene encoding serine-rich repeat protein (SRRP). Additionally, some primate isolates have other genes that are good predictors for rodent hosts. For example, the top rodent predictor based on F1 values was a magnesium transporter encoded by *corA*, which was found in the genomes of four primate strains, and the second-best rodent predictor, a putative glucose uptake protein (group_1477), was found in the genomes of 11 primate strains. It is thus likely that some rodent isolates could stably colonize the primate gastrointestinal tract and vice versa.

Logistic regression (log regression) model in *aurora* also indicated that *L. reuteri* is host-adapted (Fig 6C in the main text) but the results reveal different aspects of the adaptation. Log regression predicted more strains as weakly autochthonous. This is expected as log regression uses the most features out of all the models in *aurora*. Complex models consider a wider range of information and potentially capture more intricate patterns in the data. Thus, the sets of probabilities produced in the Threshold Calculation Phase are less granular and exhibit a high degree of variability (Fig S16) which permits many strains to be classified as weakly autochthonous. Therefore, 15 rodent isolates were predicted as either inconclusive or being associated with poultry. A closer investigation showed that even the strains labeled as inconclusive had high classification probabilities in the poultry class. The 15 strains in question have most of the top rodent predictors (*ure* operon, *corA*, SRRP) but these strains also had some predictors that are essential for classifying poultry isolates like two HTH-type transcriptional regulators (group_1722 and *bcrR*), homoserine dehydrogenase (*hom*), melibiose operon regulatory protein (*rhaS~~~rhaS_1*), threonine synthase (*thrC*) and homoserine kinase (*thrB_1~~~thrB_2*). As poultry colonization factors are not yet experimentally validated, it is not known if the strains could successfully colonize this host. However, log regression identified one poultry isolate as autochthonous in rodents. This strain indeed had all the experimentally validated rodent colonization factors (*ure* operon, SecA2-SecY2 pathway, SRRP, *glsA_1*). Despite being a poultry isolate, this strain should be able to stably colonize rodent GIT and was thus correctly labeled by *aurora*.

Results from AdaBoost highlight multiple strains whose natural habitat could not be predicted. Interestingly, AdaBoost showed that the habitat of 35% of the porcine isolates could not be identified. A closer inspection showed that this is because the strains could not be distinguished from primate isolates. In general, porcine and primate isolates are hard to distinguish as the majority of them are located in the same phylogenetic cluster (see Fig 6B in the main text) and thus share a large part of their genome. Furthermore, the distance matrix calculated from random forest proximities showed that strains isolated from primates and pigs are closely associated (Fig 6A in the main text). One of the best predictors for strains from a primate host, a surface protein G (group_2603) with F1 = 0.7, is present in all primate isolates, 24 porcine isolates out of 26, and is almost absent in all other hosts. Likewise, one of the best porcine isolates predictors, a hypothetical protein (group_4313) and ABC transporter (*expZ~~~expZ_1*) are present in 12 and 19 primate strains respectively. The results from AdaBoost also further validated results from random forest and log regression because they correctly identified primate isolates that have the *ure* operon, SecA2-SecY2 pathway, and SRRP as possible allochthonous to primates and autochthonous to rodents.

Results produced by CART indicated that seven out of a total of 27 human isolates are autochthonous in both human and poultry GIT. These seven human isolates have genes that are the best predictors for poultry hosts. For example, ribonuclease Y (group_2573) is present in 38 out of 39 poultry isolates, two rodent isolates, and seven human isolates while being absent in porcine and primate isolates. Similarly, a conductance mechanosensitive channel (*mscL_1~~~mscL*) is present in 37 out of 39 poultry isolates and in nine human isolates (seven of which were identified as autochthonous in rodent by CART) while being scarce in isolates from other hosts. Additionally, one poultry isolate was also identified as a possible autochthonous human strain. Taken together, these results show that there may be an exchange of *L. reuteri* strains between humans and poultry.

In summary, all ML algorithms implemented in *aurora* correctly predicted that *L. reuteri* is a host-adapted species, consistent with the experimental evidence. Random forest identified 7 mislabeled strains. Log regression identified 41 such strains, AdaBoost 25 and CART 18 such strains. These results are mapped to a phylogenetic tree shown in Fig 6B (see the main text). Additionally, because of their proximity to primate isolates, the 16 rodent isolates that were identified as weakly autochthonous by random forest should also be removed because they are likely to be autochthonous in two habitats. We have shown that all the algorithms are in good agreement on which strains are mislabeled or allochthonous while it is also apparent that multiple algorithms are needed to identify all the mislabeled strains or strains with adaptation to multiple hosts. Due to the number of parameters used (*e.g.,* weak regularization), log regression removed the most strains. In the subsequent GWAS analysis, we could either choose to ignore results from log regression or remove only the strains that were identified as mislabeled by all the algorithms used. The user may also fine-tune the C value which controls the regularization strength of log regression. This would result in a lower number of removed strains by log regression. We decided to ignore the results from log regression and run the GWAS analysis with only the remaining algorithms. The GWAS analysis was run in strict mode which forces the removal of all strains that were identified as mislabeled by at least one algorithm. Some strains were identified as mislabeled by multiple algorithms, so the total number of removed strains was 47 leaving 160 strains for a subsequent GWAS analysis. These strains are not randomly distributed but they tend to be located in lineages (Fig 6B in the main text).

# SUPPLEMENTARY DISCUSSION

While assessing the performance of *aurora*, we uncovered a wealth of potentially intriguing insights into the host colonization of *S.* Typhimurium. The genetic relatedness among *S.* Typhimurium strains is notably high, with the primary source of variability attributed to prophage elements [139,140]. In the first phase we compared results from *aurora* with the experimentally identified colonization factors examined in Chaudhuri *et al.* (2013) [49]. This study focused on discovery of colonization factors of only one broad-host range strain – *S.* Typhimurium SL1344. All poultry and porcine colonization factors that were discovered by Chaudhuri *et al.* (2013) were part of the core genome or they could not be mapped to our pangenome. The remaining 8 cattle-specific genes that both *aurora* and Chaudhuri *et al.* (2013) identified are all located in prophage sequences. Three genes were located in Gifsy-2 prophage: group_3917 (hypothetical protein), clpP_2~~~clpP_1 (ATP-dependent Clp protease), and group_1296 (tail assembly chaperone). Clp protease was previously described as a mouse and poultry colonization factor [49,141]. It should be noted that the pangenome of *S.* Typhimurium contains four Clp proteases. The Clp protease previously characterized as a poultry colonization factor (clpP_1, [49]) belongs to the core genome while the bovine colonization factor clpP_2~~~clpP_1 identified herein belongs to the accessory genome and is mostly present in bovine and poultry isolates. Certain aspects regarding the functionality of the Clp protease and its involvement in bovine colonization remain poorly understood [142]. Additionally to clpP_2~~~clpP_1, the Gifsy-2 prophage encodes periplasmic superoxide dismutase (*sodC*) that confers resistance to oxidative stress and is an essential mouse colonization factor [140]. Some *S.* Typhimurium strains harbor the gene outside the Gifsy-2 prophage sequence [143] and the constructed pangenome shows that the gene is present in nearly all *S.* Typhimurium strains. Identifying *sodC* as a colonization factor would thus require SNPs or k-mer targeted mGWAS. Two bovine-specific genes were found in prophage ST64B. Considerable research focus has been directed towards prophages Gifsy-1, Gifsy-2, and Gifsy-3, whereas comparatively limited attention has been directed to ST64B [139,140]. However, it is noteworthy that the prophage has been demonstrated to enhance the survival of *S.* Typhimurium in blood [144]. Lastly, two genes mapped into Gifsy-1 prophage. This prophage was previously associated with increased survival in macrophages [101].

In the next phase we examined all colonization factors that *aurora* identified to see if the results contain any of the previously experimentally verified colonization factors. With the help of clustered heatmap produced in aurora_pheno() (Fig 6A in the main text) the *S.* Typhimurium dataset was split into five clusters: poultry1, poultry2, bovine1, bovine2, and porcine1. The GWAS analysis confirmed that the two poultry clusters possess unique adaptation factors. The poultry1 cluster is phylogenetically distant from the rest of the population. Most of the colonization factors are thus lineage effects. Poultry1 is the largest cluster however the cluster is predominantly composed of clonal strains. Among the highest-ranking genes, some were found to confer resistance to mercury, a trait often linked to various antibiotic gene clusters [145]. Notably, within the top 100 genes associated with poultry1, none were the previously identified factors involved in host-bacterium interactions. Furthermore, the poultry1 strains appear to lack the virulence-associated prophages Gifsy-1 and Gifsy-2. Altogether this suggests that the poultry1 cluster might not be specifically adapted to poultry but rather represents a broad-host cluster. This hypothesis gains support from the larger size of the poultry1 genomes and the lower number of pseudogenes (Fig S19), indicating a lack of gene loss commonly observed during niche specialization [29].


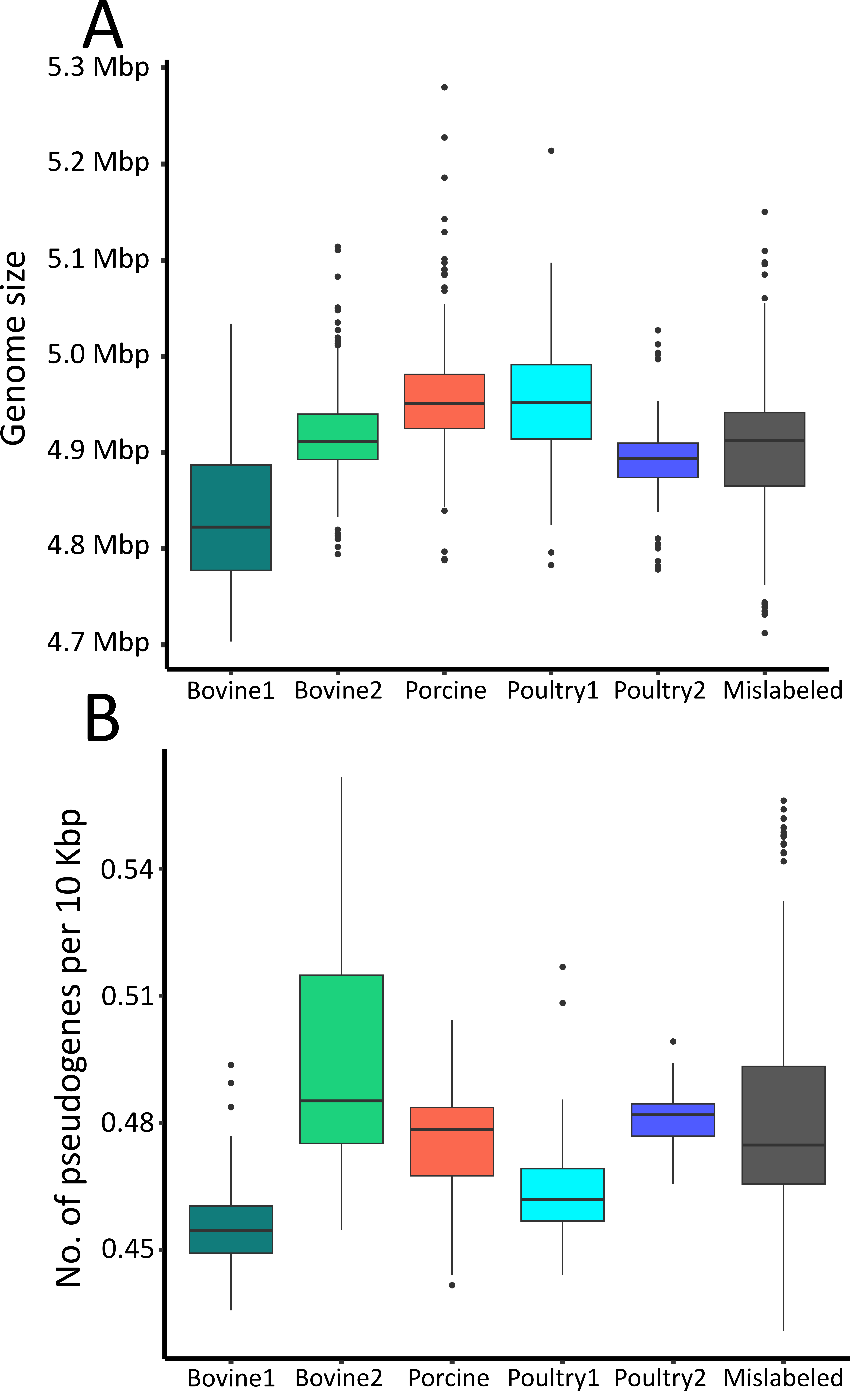


**Figure S19.** Box plots showing genome sizes A) and number of pseudogenes per 10000 bp B) of the five *S.* Typhimurium clusters identified by *aurora*. As discussed in the main text, *aurora* predicted that cluster poultry is likely not restricted only to a poultry host. Habitat specialization is often accompanied by gene loss and gene decay, both of which contribute to lower genome length and higher number of pseudogenes. This figure thus suggests that strains in poultry1 cluster did not undergo habitat specialization.

The cluster bovine1 contains a set of varied and phylogenetically distant strains. Almost 70% of these strains were classified as non-typical colonizers by *aurora*. The top bovine1 colonization factors sorted by standardized residuals or F1 values are also present in some bovine2, poultry, and porcine strains. Among the top bovine1 genes are hypothetical proteins, eukaryotic genes (likely contamination that is common in publicly available assemblies [146] or a misannotation), and housekeeping genes which should have been part of the core genome (a result of underclustering by Panaroo). Interestingly, a protein ranking 10^th^ in the bovine1 gene list is a known colonization factor *gogB* whose product inhibits pro-inflammatory host response subsequently enabling survival in macrophages [101]. The gene is not unique to bovine1 isolates but is present in some isolates from all clusters with the exception of poultry1. Another known colonization factor with nearly the same presence/absence pattern as *gogB* was ranked 12^th^ – SsrB. The two component SsrA-SsrB is important for the intracellular replication of *Salmonella* [102].

The bovine2 cluster is adapted to the host as evidenced by numerous genes with F1 values over 0.6. However, it should be noted that these genes are also present in all strains in the poultry2 cluster. There are only two genes that distinguish clusters poultry2 and bovine2: a hypothetical protein (group_1970) and an anaerobic dimethyl sulfoxide (DMSO) reductase subunit B (group_1433). These two genes are almost exclusively present in strains belonging to the poultry2 cluster. DMSO reductase is composed of three subunits (DmsABC) and allows *S.* Typhimurium to use DMSO as an anaerobic alternative electron acceptor [147]. There are at least four DMSO reductases encoded in the pangenome of *S*. Typhimurium all of which are in the core genome. It has been shown that these enzymes are functionally non-redundant and important mammalian colonization factors [147]. The gene family group_1433 encodes a periplasmic DmsB subunit which appears to be a truncated version of the four core genome *dmsB* genes. The importance of group_1433 in the colonization of poultry host was not yet investigated. Some of the poultry2-associated genes are shared with bovine2 cluster. Among these are some well-known *S.* Typhimurium colonization factors. Ranked 9^th^ was type III secretion system (T3SS) effector SseK1 and ranked 12^th^ was a NleC type T3SS effector. Both T3SS effectors play a role in *S*. Typhimurium infection [148]. Interestingly, the top 5^th^ bovine2 colonization factor was a gene encoding tricarballylate dehydrogenase (TcuA) involved in tricarballylate metabolism which was directly linked to grass tetany (metabolic disease) in ruminants [149]. Another set of pultry2 genes that ranked high are genes allowing *S*. Typhimurium to grow in the presence of arsenic. These genes are present in a subset of pultry2 isolates and are nearly absent in all other clusters. Resistance to arsenic compounds was indeed observed before in *Salmonella* strains and was hypothesized to be linked to the common usage of biocides in poultry farming [150,151].

Moreover, results from *aurora* shed light on multiple yet unknown possible colonization factors of *L. reuteri*. Amongst the top poultry colonization factors are genes nicotinamidase (*pncA*) and nicotinate phosphoribosyltransferase (*pncB*). Products of those genes are involved in NAD(+) salvage pathway. PncA was shown to be essential for the colonization of ducks by *Riemerella anatipestifer* [152] and may thus play a similar role in *L*. *reuteri* isolates. Another interesting poultry colonization factors are enzymes involved in the threonine production pathway. Indeed, increased intake of threonine in poultry was observed to increase the abundance of lactobacilli [153,154] and threonine thus seems to be a limiting factor of poultry colonization by lactobacilli. Interestingly, just as in the poultry2 cluster of *S*. Typhimurium both poultry and rodent *L. reuteri* results show that arsenate reductase, an enzyme involved in arsenic resistance, is important for the colonization of these two hosts. One of the top rodent colonization factors is a magnesium transporter. While no specific information is known about the effect of magnesium on *L*. *reuteri,* excess of intracellular magnesium limits the rodent colonization ability of multiple bacterial species [155,156]. The top rodent results also highlight the importance of Lar(MN)QO cobalt/nickel transport system. Nickel serves as a cofactor essential for the activation and functionality of urease, which is encoded by a gene cluster that is a major colonization factor [71]. Additionally, it seems like rodent isolates readily use conjugation as a means of transferring colonization factors. This is not only evidenced by the presence of conjugation associated proteins in *aurora* results (two competence factor transporting proteins, flagellar biosynthesis protein FliP and conjugal transfer pilus assembly protein TraV) but also by the number of rodent associated lineages in the *L*. *reuteri* phylogenetic tree (Fig 6B in the main text). The full list of annotated genes for all hosts is available in Additional file File S8.
